# Reversine ameliorates hallmarks of cellular senescence in human skeletal myoblasts via reactivation of autophagy

**DOI:** 10.1111/acel.13764

**Published:** 2023-01-10

**Authors:** Nika Rajabian, Debanik Choudhury, Izuagie Ikhapoh, Shilpashree Saha, Aishwarya S. Kalyankar, Pihu Mehrotra, Aref Shahini, Kendall Breed, Stelios T. Andreadis

**Affiliations:** ^1^ Department of Chemical and Biological Engineering University at Buffalo, State University of New York Amherst New York USA; ^2^ Department of Biomedical Engineering University at Buffalo, State University of New York Amherst New York USA; ^3^ Center of Excellence in Bioinformatics and Life Sciences University at Buffalo, State University of New York Amherst New York USA; ^4^ Cell, Gene and Tissue Engineering (CGTE) Center, School of Engineering and Applied Sciences University at Buffalo, State University of New York Amherst New York USA

**Keywords:** aging, cellular senescence, metabolism, methionine pathway, skeletal muscle

## Abstract

Cellular senescence leads to the depletion of myogenic progenitors and decreased regenerative capacity. We show that the small molecule 2,6‐disubstituted purine, reversine, can improve some well‐known hallmarks of cellular aging in senescent myoblast cells. Reversine reactivated autophagy and insulin signaling pathway via upregulation of Adenosine Monophosphate‐activated protein kinase (AMPK) and Akt2, restoring insulin sensitivity and glucose uptake in senescent cells. Reversine also restored the loss of connectivity of glycolysis to the TCA cycle, thus restoring dysfunctional mitochondria and the impaired myogenic differentiation potential of senescent myoblasts. Altogether, our data suggest that cellular senescence can be reversed by treatment with a single small molecule without employing genetic reprogramming technologies.

## Graphical abstract

Our results suggest that short‐term treatment of senescent myoblasts with reversine could restore insulin resistance, enhance glucose metabolism and oxidative phosphorylation, likely via reactivation of autophagy. Restoring DNA damage and the state of heterochromatin preceded restoration of proliferation, SA‐β‐Gal expression, and cell size in reversine‐treated cells, ultimately restoring the differentiation ability of myoblasts to form myofibers. Therefore, reversine may have the potential to be used as a novel, anti‐aging treatment, without the tumorigenic complications of genetic reprogramming technologies.

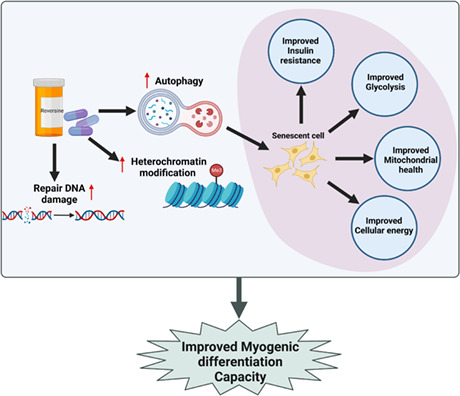

**Abbreviations**
2‐DG: 2‐deoxy‐D‐glucose
AMPK: adenosine monophosphate‐activated protein kinase
ATP: adenosine triphosphate
B2M: beta‐2‐microglobulin
CC: compound C
CQ: chloroquine
DMSO: dimethyl sulfoxide
ECAR: extraCellular acidification rate
eSM: etoposide treated senescent myoblast
GA: glucose analog
GAPDH: glyceraldehyde‐3‐phosphate dehydrogenase
GM: growth medium
H3K9me3: tri‐methylation of histone 3 at Lys9
H3K27me3: tri‐methylation of histone 3 at Lys27
LC3: light chain 3
mtDNA: mitochondrial DNA
nDNA: nuclear DNA
OCR: oxygen consumption rate
PDH: pyruvate dehydrogenase
PINK1: PTEN‐induced kinase 1
SA‐β‐Gal: senescence‐associated beta galactosidase
SM: senescent myoblast
TCA cycle: tricarboxylic acid cycle
TMRM: tetramethylrhodamine, methyl ester
YM: young myoblast

## 1 INTRODUCTION

Aging results in gradual loss of muscle function, which is associated with reduction in muscle mass and strength (Etienne et al., 2020). Age‐related loss of muscle mass may decrease mobility and increase the risk of morbidities and mortality. As of 2010, 524 million people around the world were 65 years of age or older and by 2050, experts expect this number to grow to 1.5 billion (Morgan et al., 2016). This is a major health issue given that aging is accompanied by increased need for healthcare, long‐term care and social services to support older adults.

Skeletal muscle has a remarkable capacity to regenerate by activation of myogenic progenitor cells; however, both the number of these progenitors and their regenerative capacity decline with aging and cellular senescence (Muñoz‐Cánoves et al., 2020; Yamakawa et al., 2020). Recent studies showed that DNA damage and epigenetic alterations are primary hallmarks of aging leading to dysregulated nutrient sensing, mitochondrial dysfunction, and ultimately loss of muscle function (López‐Otín et al., 2013). Metabolic changes such as impaired glycolysis, insulin sensitivity, and mitochondrial respiration are affected by senescence contributing to loss of the myoblast capacity to differentiate (Baraibar et al., 2016; Pääsuke et al., 2016; Ravera et al., 2019; Shou et al., 2020; Trifunovic & Larsson, 2008). Aging is also associated with impaired autophagy, which is essential to maintain satellite cell stemness and mitochondrial turn over (García‐Prat et al., 2016; Tang & Rando, 2014). Cellular reprogramming using the four Yamanaka factors (Oct4, Sox2, Klf4, and c‐Myc) has been shown to ameliorate the aging hallmarks in somatic cells (Ravaioli et al., 2018; Strässler et al., 2018; Yener Ilce et al., 2018), including skeletal muscle (Wang et al., 2021) and mesenchymal stem cells supporting wound regeneration (Kurita et al., 2018). However, genetic reprogramming can lead to teratoma formation (de Lázaro et al., 2017; Ohnishi et al., 2014; Tamanini et al., 2018; Wang et al., 2021), thereby posing significant concerns as a rejuvenation strategy.

Several studies found that the small molecule 2,6‐disubstituted purine, reversine, increased cellular plasticity as demonstrated by increased differentiation potential of progenitor cells toward the neuroectodermal lineage (Lee et al., 2009); dedifferentiation of C2C12 myoblasts to a progenitor‐like state (Chen et al., 2004, 2007); as well as dedifferentiation of sheep fibroblasts into multipotent progenitor cells, possibly via expression of the pluripotent factor, Oct4 (Guo et al., 2021). These progenitors could be induced to differentiate into adipocytes, osteoblasts, hepatocytes, and neurocytes in vitro (Guo et al., 2021). Other studies reported that reversine may have anticancer properties (D'Alise et al., 2008; Piccoli et al., 2020). Indeed, reversine is an Aurora B protein kinase inhibitor, causing failure in mitotic chromosome segregation, cytokinesis, and cell proliferation (Amabile et al., 2009; D'Alise et al., 2008). Besides the inhibition of Aurora B kinase, reversine was found to induce autophagic cell death of cancer cells (Fang et al., 2018; Lu et al., 2012; Prajumwongs et al., 2021).

Based on these results, we hypothesized that reversine might ameliorate the hallmarks of cellular senescence in human myoblasts. We discovered that short‐term treatment of fully senescent myoblasts with reversine could restore insulin resistance, enhance glucose metabolism and oxidative phosphorylation, likely via reactivation of autophagy, ultimately restoring the differentiation ability of myoblasts to form myofibers. Our results suggest that reversine may have the potential to be used as a novel, antiaging treatment, without the tumorigenic complications of genetic reprogramming technologies.

## 2 RESULTS

### 2.1 Reversine improves hallmarks of cellular senescence in human myoblasts

Recently, our laboratory showed that human myoblasts that were cultured for 20 population doublings (equivalent to >50 days of culture, replicative senescence) exhibited all major hallmarks of senescence including expression of SA‐β‐gal, increased size, flattened morphology, reduced proliferation, DNA damage, histone modifications, and inability to form myotubes (Rajabian et al., 2020; Shahini et al., 2021). Immunostaining for desmin and MyoD (a myoblast marker) was performed in young and senescent myoblasts from all three donors (Figure S1a–c). The vast majority of young and senescent myoblasts expressed desmin (94.8%–97.5%). The percentage of cells positive for MyoD was high in young myoblasts (73.5%–79.8%), and decreased significantly in senescent myoblasts (9.9%–14.9%). The decline in the % of MyoD+ in senescent cells is in agreement with the impaired proliferation and myogenic differentiation capacity.

The doubling times of young myoblasts from all donors (18yrM: 42.1 ± 6, 25yrF: 52.3 ± 6, 75yrF: 46.3 ± 1 h) were significantly shorter than those of senescent myoblasts (18yrM: 89.7 ± 9, 25yrF: 89.2 ± 5, 75yrF:84.3 ± 11 h) (Figure S1d). In addition, senescent myoblasts from all three donors exhibited significant loss of myogenic differentiation capacity, as evidenced by the measures of myotube diameter and fusion index (Figure S1e,f) (*Myotube diameter*: 18yrM, YM: 95 ± 7 μm, SM: 25 ± 1 μm; 25yrF, YM: 92 ± 6 μm, SM: 22 ± 2 μm; 75yrF, YM: 101 ± 6 μm, SM: 23 ± 2 μm; *Fusion Index*: 18yrM, YM: 63 ± 2%, SM: 15 ± 1%; 25yrF, YM: 61 ± 2%, SM: 13 ± 2%; 75yrF, YM: 63 ± 5%, SM: 14 ± 2%).

Furthermore, we evaluated some hallmarks of senescence such as expression of senescence‐associated‐β‐galactosidase, DNA damage (γH2AX), and histone modifications (H3K9me3 and H3K27me3). Each dot in the bar graph (Figure S1g–j) indicates one donor. The percentage of young myoblasts that stained positive for SA‐β‐Gal varied between different donors (18yrM: 7.3%, 25yrF: 11.5%, 75yrF: 15.1%) but was significantly lower than that of senescent myoblasts (18yrM: 61.8%, 25yrF: 78.4%, 75yrF: 72.8%) (Figure S1g). The level of γH2AX was significantly higher in senescent myoblasts (18yrM: ~1.5 fold change, 25yrF: ~3.2 fold change, 75yrF: ~1.74 fold change) as compared to young myoblasts cells (Figure S1h). We also observed decreased levels of H3K9me3 and H3K27me3 in senescent myoblasts as compared to young ones (H3K9me3; 18yrM: ~2.3 fold change, 25yrF: ~1.57 fold change, 75yrF: ~2.1 fold change, H3K27me3; 18yrM: ~3.1 fold change, 25yrF: ~1.81 fold change, 75yrF: ~1.8 fold change) (Figure S1i,j).

**FIGURE 1:**
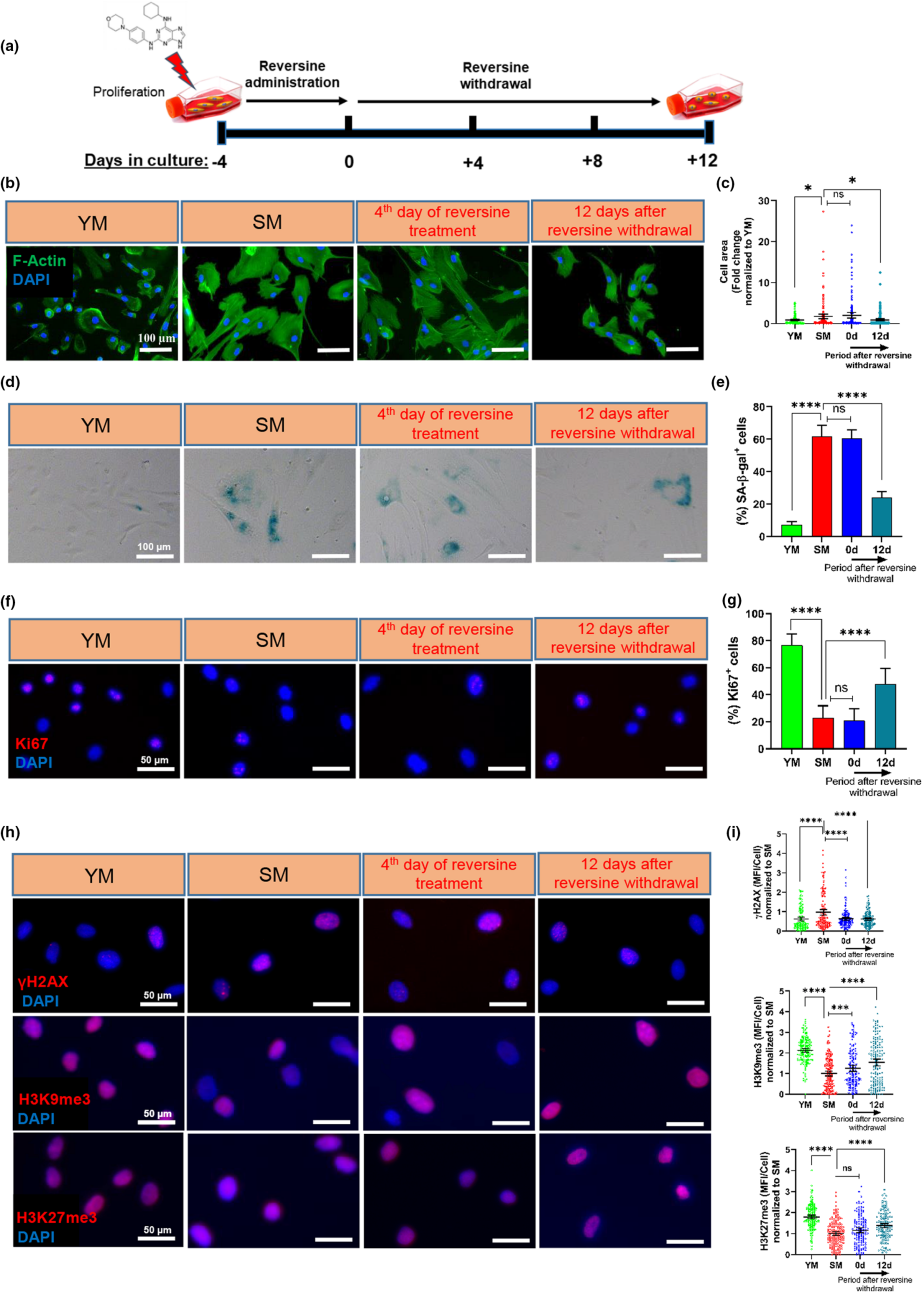
Improvements of senescence hallmarks in human myoblasts upon reversine treatment. (a) Schematic illustration of the experimental design: Senescent myoblast cells were treated with reversine for 4 days, reversine was removed and various measurements were performed at 0, 4, 8 or 12 days post‐treatment. (b and c) Immunostaining for F‐Actin to show morphology and cells size in young, senescent and reversine‐treated myoblasts at 0 or 12 days post‐treatment (scale bar = 100 μm, *n* = 150 cells). (d and e) Staining for SA‐β‐gal in young, senescent, 0 and 12 days myoblast cells and quantification of the percentage of SA‐β‐gal‐positive cells (*n* = 250 cells) (scale bar = 100 μm). (f and g) Immunostaining for Ki67 and quantification of the percentage of Ki67+ cells in young, senescent, 0 and 12 days myoblast cells (scale bar = 50 μm, *n* = 150 cells). (h and i) Immunostaining and quantification for γH2AX, H3K9me3 and H3K27me3 on 0 and 12 days cells (scale bar = 50 μm, *n* = 150 cells). Data in bar graphs are presented as mean ± SD and data in dot plots are presented as mean ± 95% confidence interval. * denotes *p* < 0.05, ** denotes *p* < 0.01, *** denotes *p* < 0.001, **** denotes *p* < 0.0001 and ns, not significant.

In this study, we examined whether the small molecule 2,6‐disubstituted purine or reversine could reverse the hallmarks of replicative senescence in human myoblasts in vitro. To this end, senescent human myoblast (SM) cells were treated with reversine or DMSO (control) for 4 days, and cellular morphology was determined at different times after treatment using F‐actin staining (Figure 1a). The area of senescent myoblasts was much larger, and their morphology appeared flattened as compared to young myoblasts. While there was no significant effect on the shape or size immediately after treatment (*t* = 0 days), by 12 days after reversine withdrawal, cell size decreased significantly as compared to senescent myoblasts (Figure 1b,c). By Day 8, many smaller cells appeared in culture and by 12 days after withdrawal, the majority of cells were smaller than the starting senescent cells (Figure S2a). Immunostaining showed that small cells stained positive for the myoblast marker desmin, indicating that they retained their myoblast phenotype (Figure S2a,b). We also examined whether longer treatment with reversine would be even more effective. Our results showed that there was no significant difference between 4 days and 12 days of reversine treatment with regard to the levels of desmin, γH2AX, and H3K9me3 expressions (Figure S1k–n). Therefore, we chose the 4‐day treatment for the rest of our experiments.

As senescence‐associated β‐galactosidase (SA‐β‐Gal) is a well‐known marker of cellular senescence (Debacq‐Chainiaux et al., 2009), we measured the percentage of SA‐β‐Gal+ cells. The % of SA‐β‐Gal+ cells in senescent myoblast cultures was higher as compared to young myoblasts (Figure 1d,e; YM: 7.3 ± 1.7%, SM: 61.8 ± 6.7%). The % SA‐β‐Gal^+^ cells did not change significantly immediately after treatment (*t* = 0 days) but decreased dramatically 12 days after reversine withdrawal (Figure 1d,e; 0 days: 60.6 ± 5.2%, 12 days: 23.9 ± 3.8%).

As expected, the cumulative cell number of senescent myoblast cells decreased as compared to young myoblasts. Similar to cell size and SA‐β‐gal expression, the cell proliferation started to increase after 8 days of reversine withdrawal and continued for 40 days after reversine withdrawal, when the cumulative cell number was significantly higher (19.1 ± 1.8 fold change) than that of senescence myoblasts (Figure S2c). As expected, immunostaining for the proliferation marker, Ki67, showed that the % Ki67^+^ senescent myoblasts was significantly lower than in young ones (Figure 1f,g; YM: 76.5 ± 7.8%, SM: 22.8 ± 8.6%). However, 12 days after reversine treatment, the %Ki67^+^ cells increased significantly (Figure 1f,g; 0 days: 20.7 ± 8.4%, 12 days: 47.8 ± 11.2%), in agreement with the increased cell number.

Finally, we examined the effects of reversine on senescence‐associated DNA damage and heterochromatin modifications (López‐Otín et al., 2013). Interestingly, reversine treatment for 4 days reduced the expression level of γH2AX, a marker of DNA damage, in senescent myoblast cells and the low γH2AX levels persisted 12 ‐day post‐treatment (Figure 1h,i). In agreement with previous reports (Ocampo et al., 2016; Rajabian et al., 2020), we also observed decreased heterochromatin in senescent myoblasts as shown by immunostaining for heterochromatin marks H3K9me3 and H3K27me3. The level of H3K9me3 increased significantly with 4 days of reversine treatment and remained high after 12 days. On the contrary, the level of H3K27me3 did not change immediately after treatment but increased significantly 12 days later (Figure 1h,i). These results suggested that reversine may be used to ameliorate hallmarks of senescence in aged SM and prompted us to examine its potential effects on cellular metabolism.

As reversine was dissolved in DMSO, we examined whether treatment with DMSO alone for 4 days had any effects on senescent myoblasts. Although no effects were seen immediately after treatment (0 days), on 12 days after DMSO withdrawal, we observed small but significant increase in %SA‐b‐gal^+^ cells (Figure S3a,c; SM: 60.5 ± 9.4%, 0 days: 63.2 ± 8.5%, 12 days: 79.3 ± 8.5%), increased level of γH2AX (Figure S3a,e), decreased %Ki67^+^ cells (Figure S3a,d; SM: 18.9 ± 3.9%, 0 days: 18.5 ± 4.7%, 12 days: 14.3 ± 4.8%) and decreased levels of H3K9me3 and H3K27me3 (Figure S3a,f,g), all of them in opposite direction to reversine treatment. Therefore, DMSO appears to slightly counteract the effects of reversine, suggesting that the effects of reversine might be underestimated.

### 2.2 Impaired glycolytic capacity is restored in reversine‐treated cells

**FIGURE 2:**
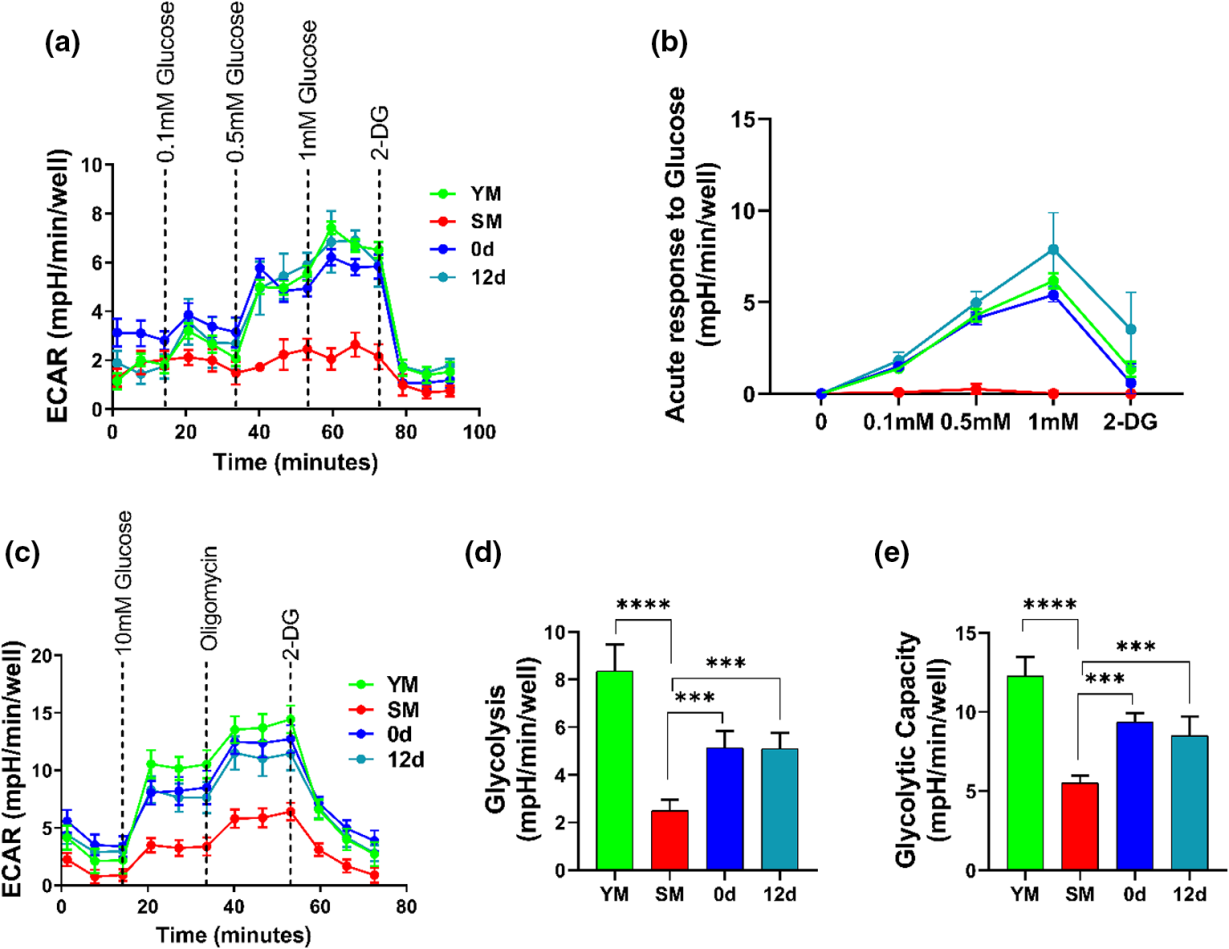
Impaired glycolytic capacity is restored by reversine. (a) Measurements of extracellular acidification rate with different dosage of glucose. (b) Acute response indicating glucose sensitivity. (c–e) Measurements of extracellular acidification rate and calculation of glycolysis and glycolytic capacity from ECAR. Data in ECAR plot is presented as mean ± SEM. *** denotes *p* < 0.001, **** denotes *p* < 0.0001.

Previous studies demonstrated that aging is associated with impairment of glycolysis (Baraibar et al., 2016; Pääsuke et al., 2016; Ravera et al., 2019), prompting us to examine the effect of reversine treatment on glucose metabolism. To this end, we employed Seahorse analysis to measure the ECAR (ExtraCellular Acidification Rate), a measure of lactate secretion. Interestingly, increasing dosage of glucose gradually increased ECAR in young myoblast and reversine‐treated cells (immediately after treatment, 0 and 12 days after reversine withdrawal) but not in senescent myoblasts (Figure 2a,b).

Furthermore, we investigated the effect of reversine on glycolysis and glycolytic capacity using the Seahorse glycolysis stress test. In agreement with the previous result, glucose injection induced greater ECAR in young and reversine‐treated myoblasts (0 and 12 days) as compared to senescent myoblasts. In addition, young and reversine‐treated senescent myoblasts (0 and 12 days) were more responsive to oligomycin (ATP synthase inhibitor, IC50 = 1 μM) than senescent myoblasts. As a result, glycolysis and glycolytic capacity were significantly lower in senescent myoblast cells but were partially restored by reversine (Figure 2c–e). In agreement with restoring glycolysis, young and reversine‐treated senescent cells (0 and 12 days) demonstrated a dose‐dependent decrease in ATP in response to 2‐DG (hexokinase inhibitor, IC50 = 5 mM), which was not observed in senescent myoblasts (Figure S4a). These results suggested that reversine could restore glycolysis, prompting us to investigate this pathway further.

### 2.3 Reversine treatment improves insulin resistance in senescent myoblasts

**FIGURE 3:**
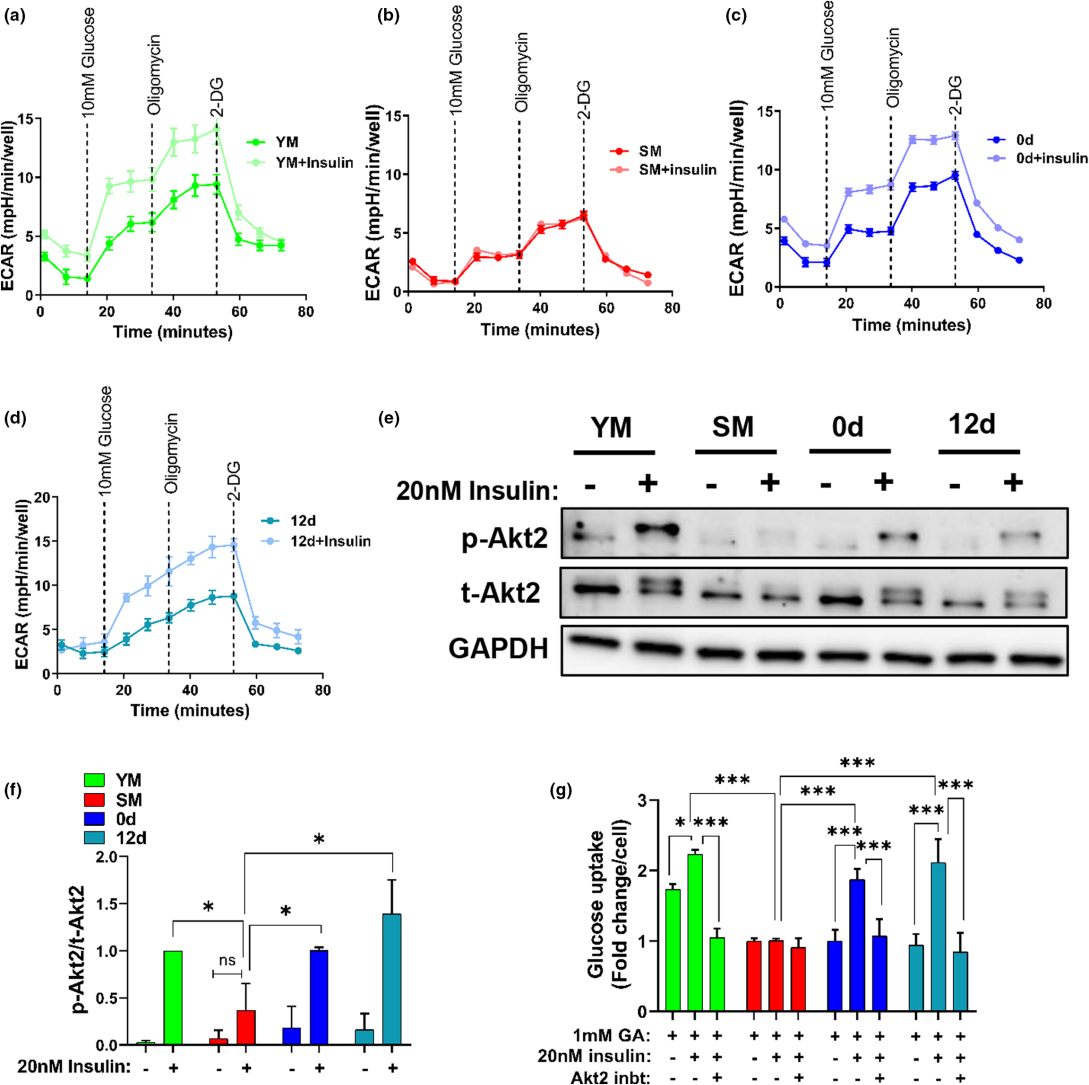
Reversine treatment improves insulin resistance in senescent myoblasts. Measurements of extracellular acidification rate in young, senescent control and reversine‐‐treated myoblasts at 0 or 12 days post‐treatment. Each condition was done with or without 20 nM insulin for 30 min using Seahorse extracellular flux analyzer. (e) Western blots of total (t) and phosphorylated (p)Akt2; GAPDH served as a loading control. (f) Quantification of Western blot showing the ratio of p‐Akt2 to t‐Akt2 in young, senescent, 0 and 12 days myoblast cells. (g) Measurement of glucose uptake in response to insulin in the presence or absence of Akt2 inhibition. Data in ECAR plot are presented as mean ± SEM and data in bar graphs are presented as mean ± SD. * denotes *p* < 0.05, ** denotes *p* < 0.01, *** denotes *p* < 0.001, and ns. not significant.

Since age‐impaired glucose homeostasis is associated with insulin resistance and defects in insulin signaling in skeletal muscle (Refaie et al., 2006; Shou et al., 2020), we investigated the effect of reversine treatment on insulin sensitivity, by measuring ECAR after treating the cells with insulin. Insulin induced greater ECAR in young and interestingly, also in reversine‐treated cells (0 and 12 days), but had no effect on senescent myoblasts (Figure 3a–d). This result prompted us to investigate the serine/threonine kinase Akt (protein kinase B) pathway, which has been implicated in insulin‐stimulated glucose uptake (Cho et al., 2001; Jaiswal et al., 2019; McCurdy & Cartee, 2005). To this end, we examined the expression of total (t)‐Akt2 and the capacity of insulin to induce phosphorylation, (p)‐Akt1/2. All four groups expressed t‐Akt2, which was phosphorylated by insulin (20 nM) in young myoblast and reversine‐treated cells (0 and 12 days) but not senescent myoblast cells (Figure 3e,f). At the same time, all four groups expressed t‐Akt1; the level of phosphorylation of Akt1 was not affected immediately after reversine treatment (0 days) but increased significantly 12 days after reversine withdrawal (Figure S5a).

As Akt2 is essential in insulin resistance (Cho et al., 2001), we further tested whether the inhibition of Akt2 signaling could suppress insulin‐dependent glucose uptake. To this end, we measured glucose uptake using 1 mM of a glucose analog (GA) that fluoresces upon phosphorylation of the achiral carbon. Glucose uptake increased upon insulin stimulation of young and reversine‐treated myoblasts (0 and 12 days) and decreased significantly in the presence of the Akt2 selective inhibitor (CCT128930, IC50 = 5 μM) (Figure 3g). By contrast, glucose uptake was low and was not affected by insulin treatment or Akt2 inhibition in senescent myoblasts, demonstrating that senescent cells developed insulin resistance, which was restored by reversine. Furthermore, Akt2 inhibition decreased the stimulatory effect of insulin on ATP in young and reversine‐treated myoblasts (0 and 12 days) but not in senescent cells (Figure S5b).

### 2.4 Reversine induces autophagy and decreases the size of senescent myoblasts

**FIGURE 4:**
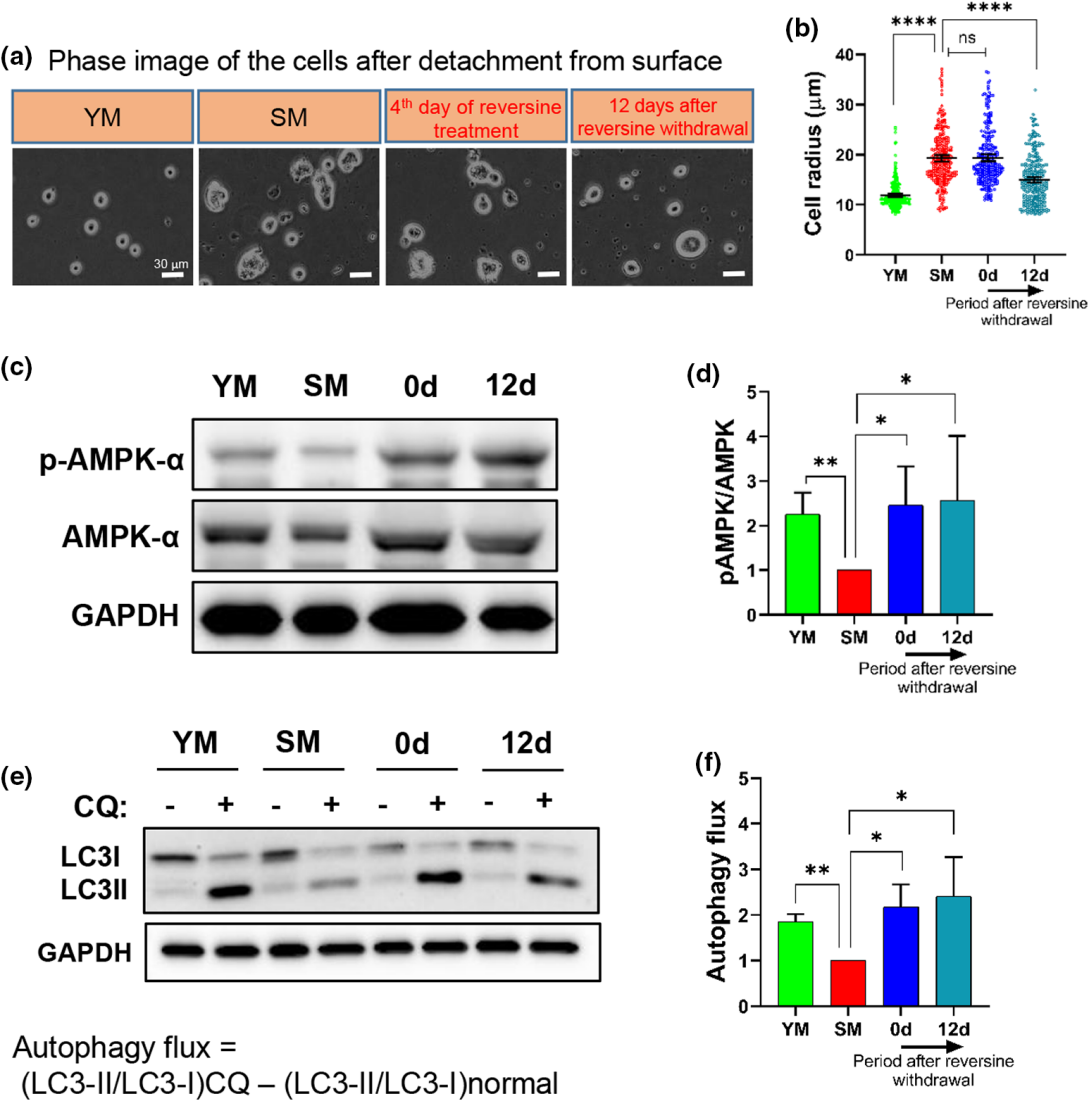
Reversine induces autophagy and decreases the size of senescent myoblasts. (a) Phase images of the myoblasts detached from the surface. (b) Quantification of cell radius; data shown as means ±95% CI (*n* = 250 cells) (scale bar = 30 μm). (c and d) Western blotting analysis for quantifying the total protein and phosphorylation of AMPKα (Thr172). GAPDH served as a loading control. (e and f) Western blotting analysis of LC3 protein upon starvation+chloroquine treatment for 6 h and quantification of the autophagy flux using the formula: Autophagy flux = (LC3‐II/LC3‐I)_CQ_ − (LC3‐II/LC3‐I)_Control_. Data in ECAR plot are presented as mean ± SEM. Data in bar graphs are presented as mean ± SD. * denotes *p* < 0.05, ** denotes *p* < 0.01, **** denotes *p* < 0.0001 and ns, not significant.

As shown above, reversine significantly decreased senescence‐associated cell size (Figure 4a,b), suggesting that autophagy might be a contributing factor (Hosokawa et al., 2006; Miettinen & Björklund, 2015). To address this hypothesis, we measured the phosphorylation of adenosine monophosphate‐activated protein kinase α (AMPKα) at Thr172, which activates AMPKα and positively regulates autophagy (Jeon, 2016). Immunoblots revealed that phosphorylation of AMPKα (pAMPKα) was significantly decreased in senescent myoblasts but was restored in reversine‐treated cells (0 and 12 days) (Figure 4c,d).

To evaluate the effect of reversine on autophagy, we measured autophagosome formation by starving the cells for 6 h, while inhibiting autophagosome‐lysosome fusion by chloroquine (CQ, (starvation+chloroquine) condition). Western blot for the autophagosome marker, microtubule‐associated protein (MAP) light chain 3 (LC3) showed that the autophagy flux decreased in senescent myoblasts but was restored by reversine to the level of young myoblasts (0 and 12 days) (Figure 4e,f).

### 2.5 Reversine improves mitochondrial function in senescent myoblast cells

**FIGURE 5:**
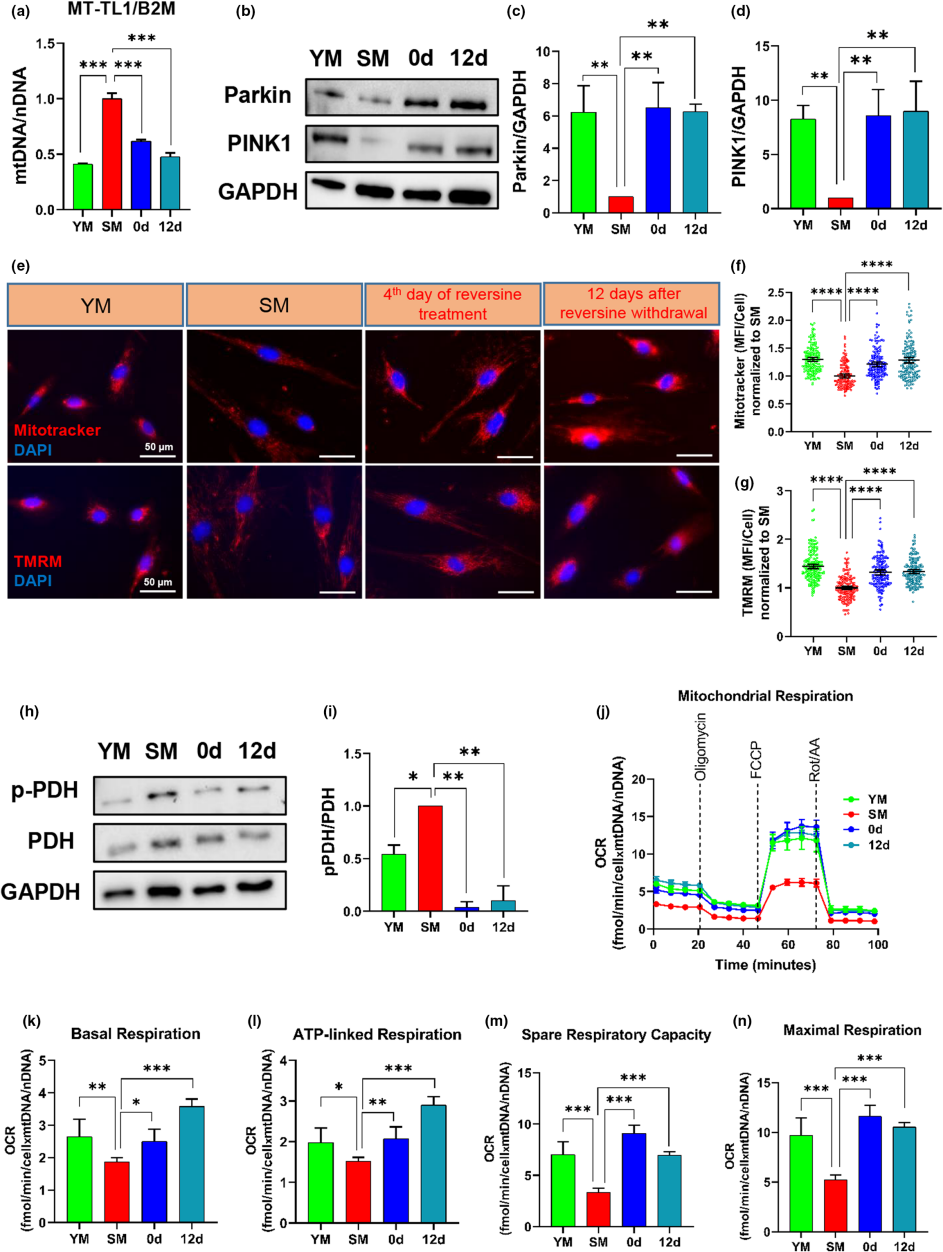
Reversine improves mitochondrial function in senescent myoblasts. (a) Quantitative real‐time PCR quantification of mtDNA relative to nDNA (mtDNA/nDNA) as measured by primers specific to mitochondrial gene MT‐TL1 and nuclear gene B2M in young, senescent, 0 and 12 days myoblast cells. (b–d) Western blotting and quantification of parkin and PINK1 proteins. (e) Representative images of tetramethylrhodamine methyl ester (TMRM) and MitoTracker live stains corresponding to mitochondrial membrane potential (scale bar = 50 μm). (f and g) Quantification of TMRM and MitoTracker intensity per cell; data shown as means ±95% CI (*n* = 150 cells). (h) Western blots of total (t) and phosphorylated (p)PDH; GAPDH served as a loading control. (i) Quantification of western blot showing ratio of p‐PDH to PDH in young, senescent, 0 and 12 days myoblast cells. (j) Measurements of oxygen consumption rate (OCR) using Seahorse extracellular flux analyzer. (k‐n) Calculations of the basal, maximal, reserved and ATP‐linked respiration rates. Data in OCR plot is presented as mean ± SEM and data in bar graphs are presented as mean ± SD. * denotes *p* < 0.05, ** denotes *p* < 0.01, *** denotes *p* < 0.001 and **** denotes *p* < 0.0001.

Senescence is associated with mitochondrial DNA (mtDNA) damage, mitochondrial dysfunction and decline in respiratory chain function (Trifunovic & Larsson, 2008). To examine the effects of reversine on mitochondrial function, first we measured the ratio of mitochondrial over nuclear DNA (mtDNA/nDNA) using qPCR. As expected, the level of mtDNA/nDNA was higher in senescent as compared to young myoblast cells, but was restored by reversine treatment (Figure 5a). Furthermore, senescent myoblasts had lower levels of Parkin and PINK1, both of which were restored by reversine (Figure 5b–d). PINK1 senses mitochondria damage and accumulates on the outer membrane of damaged mitochondrial where it recruits the ubiquitin ligase Parkin to induce mitophagy (Bingol & Sheng, 2016; Truban et al., 2017). Therefore, the increased level of PINK1 and Parkin suggest restoration of mitophagy that was diminished in senescent myoblasts. Similarly, immunostaining for TMRM and MitoTracker Red CMXRos fluorescent probes showed that the mitochondrial membrane potential was significantly lower in senescent myoblasts but was also restored by reversine (Figure 5e–g), suggesting that reversine might restore the impaired mitochondrial function of senescent myoblasts.

Since reversine restored glycolysis and mitochondrial function, we examined whether it also affected the pyruvate dehydrogenase complex, which catalyzes the formation of acetyl‐CoA from pyruvate, linking glycolysis to oxidative phosphorylation (Patel et al., 2014). As expected, we found higher levels of phosphorylated (p)‐PDH in SM cells (Figure 5h,k), suggesting loss of connectivity of glycolysis to the TCA cycle. Interestingly, pPDH decreased by reversine (0 and 12 days) to even lower levels than in young myoblasts (Figure 5h,i). This result suggested that mitochondrial respiration and ATP production could also be fully restored by reversine. Indeed, measurements of OCR showed that the basal, maximal, reserved, and ATP‐linked respiration capacity were all restored in reversine‐treated cells (0 and 12 days) (Figure 5j‐n), suggesting that reversine increased oxidative phosphorylation and mitochondrial respiration.

### 2.6 Reversine improves metabolic changes in senescent myoblasts by reactivating autophagy

**FIGURE 6:**
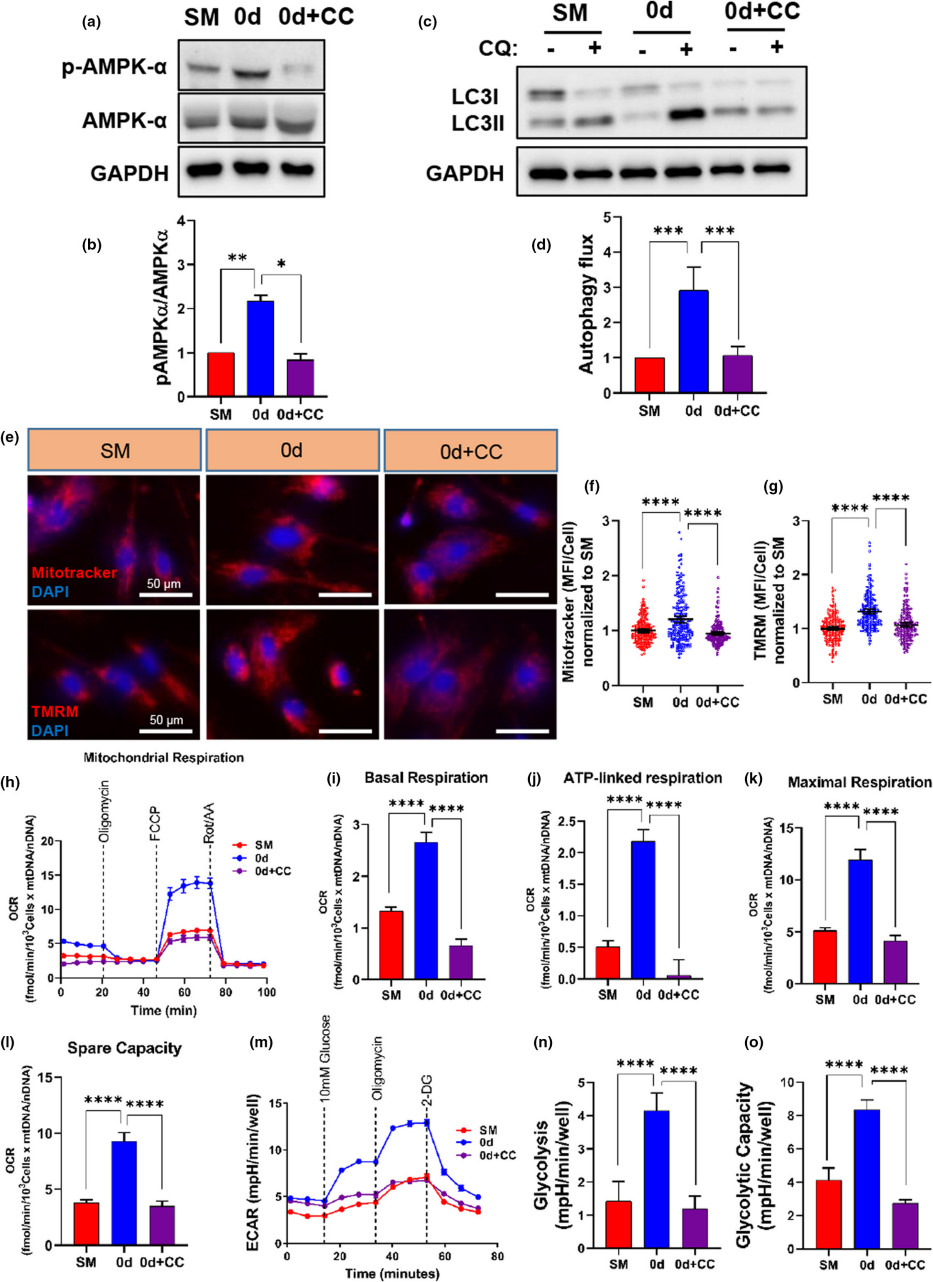
Reversine improves the metabolism of senescent myoblast cells by reactivating autophagy. (a, and b) Western blotting analysis for total and phosphorylated AMPKα (Thr172). GAPDH served as a loading control. (c and d) Western blotting analysis of LC3 protein upon starvation+chloroquine treatment for 6 h and quantification of the autophagy flux using the formula: Autophagy flux = (LC3‐II/LC3‐I)CQ – (LC3‐II/LC3‐I)control. (e–g) Representative images and quantification of tetramethylrhodamine methyl ester (TMRM) and MitoTracker live staining (scale bar = 50 μm); data shown as means ±95% CI (*n* = 150 cells). (h) Measurements of oxygen consumption rate (OCR) using seahorse extracellular flux analyzer. (i–l) Calculations of the basal, maximal, reserved and ATP‐linked respiration rates. (m) Measurements of extracellular acidification rate and (n and o) calculation of glycolysis and glycolytic capacity from ECAR. Data in OCR and ECAR plot are presented as mean ± SEM and data in bar graphs are presented as mean ± SD. * denotes *p* < 0.05, ** denotes *p* < 0.01, *** denotes *p* < 0.001 and **** denotes *p* < 0.0001.

The reversal of pAMPKα/AMPKα levels prompted us to hypothesize that reversine might improve mitochondrial function and glucose metabolism by restoring autophagy. To address this hypothesis, we inhibited AMPK in reversine‐treated cells by the addition of compound C (CC; 1 μg/ml during the 4 days of reversine treatment), which inhibits AMPK by occupying a pocket that partially overlaps with the ATP active site in the AMPKα subunit (Handa et al., 2011). Western blots for AMPKα and pAMPKα showed that CC decreased pAMPKα/AMPKα to a similar level as that of senescent myoblasts (Figure 6a,b). As a result, the autophagy flux decreased significantly with inhibition of AMPKα phosphorylation in reversine‐treated cells (Figure 6c,d). AMP‐activated protein kinase inhibition also abrogated the effects of reversine on mitochondrial membrane potential as measured by the intensity of MitoTracker and tetramethylrhodamine methyl ester (TMRM) (Figure 6e–g) and affected cellular metabolism significantly. Specifically, CC reduced the oxygen consumption rate (OCR) and reversed the effects of reversine on basal, ATP‐linked, maximal respiration and spare respiratory capacity; as well as extra cellular acidification rate (Decary et al., 1997), reversing the effects of reversine on glycolysis and glycolytic capacity (Figure 6h–o). These results suggest that reversine may be reversing the loss of metabolic function of senescent cells by activating autophagy.

### 2.7 Reversine restores myogenic differentiation potential in senescent myoblast cells

**FIGURE 7:**
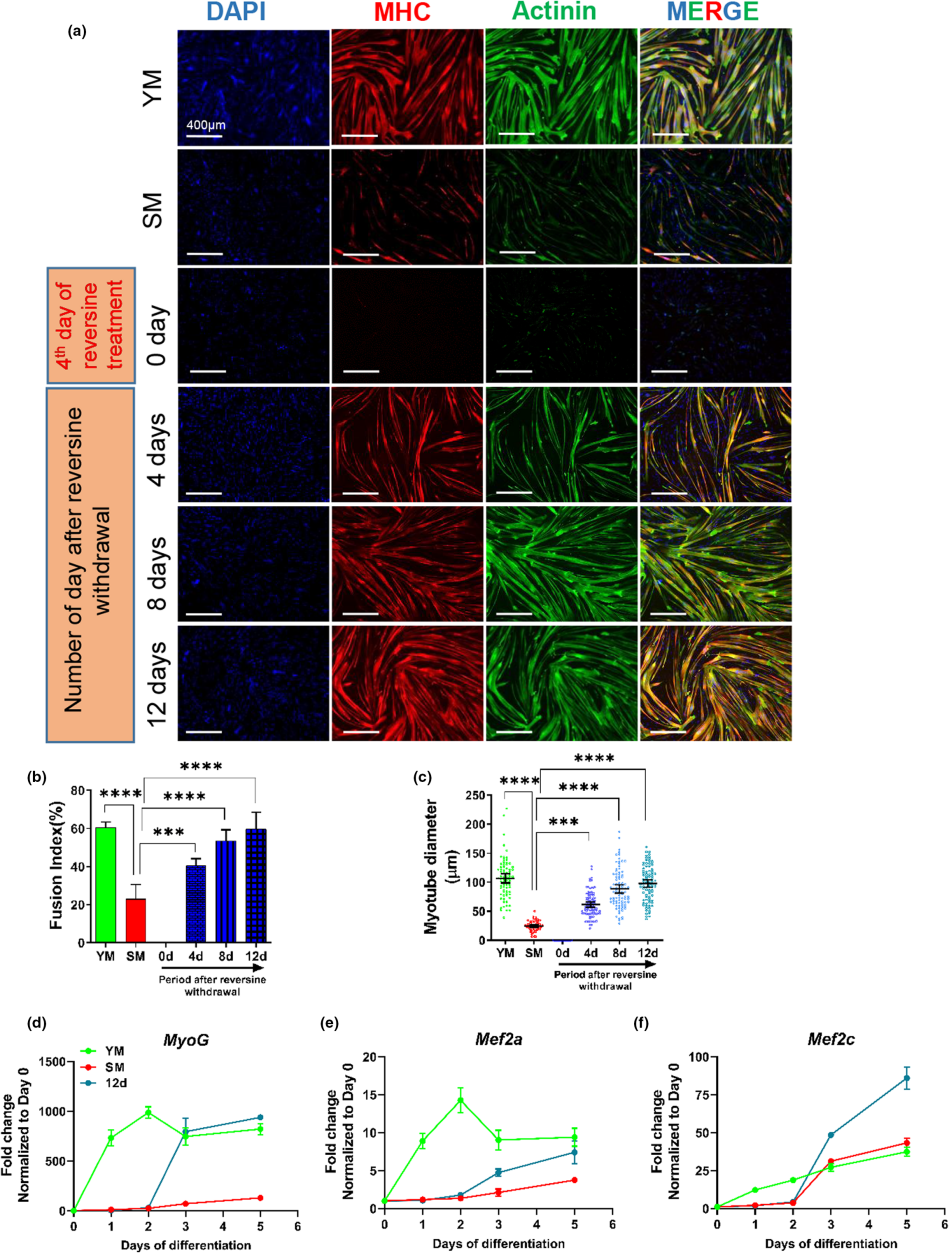
Reversine restores myogenic differentiation potential in senescent myoblasts. (a) Immunostaining for MHC (red) and Actinin of myotubes formed by young, senescent control and reversine‐treated myoblasts. Nuclei were stained with DAPI (blue). Differentiation started on 0, 4, 8, or 12 days after reversine withdrawal (scale bar = 400 μm). (b) Quantification of the fusion index (number of myonuclei/total number of nuclei × 100%) and (c) the average diameter of myotubes generated by young, senescent control, and reversine‐treated myoblasts, where differentiation started on 0, 4, 8 or 12 days after reversine withdrawal. (d–f) Quantitative real‐time PCR quantification of gene expression of *MyoG, Mef2a* and *Mef2c* during myogenic differentiation. Data in bar graphs are presented as mean ± SD and data in dot plots are presented as mean ± 95% confidence interval. *** denotes *p* < 0.001 and **** denotes *p* < 0.0001.

Since reversine improved some well‐known hallmarks of cellular senescence as well as glycolysis and mitochondrial function, we examined whether it could also restore the impaired myogenic differentiation potential of senescent myoblasts. In line with previous studies (Bigot et al., 2008; Rajabian et al., 2020), we observed that replicative senescence impaired the ability of myoblasts to form myotubes (Figure 7a). The myotube diameter and the fusion index (FI, defined as the percentage of nuclei in myotubes) decreased significantly in senescent as compared to young myoblasts (Figure 7a–c; myotube diameter, YM: 106 ± 34 μm, SM: 24 ± 8 μm; FI, YM: 60 ± 2%, SM: 23 ± 5%, *n* = 3, *p* < 0.0001 as compared to SM). Interestingly, reversine treatment for 4 days decreased MyoD and Mef2c, known as key regulators of skeletal muscle myogenesis, in senescent myoblasts (Figure S6a–c), in agreement with decreased myotube formation after 4 days of reversine treatment. However, upon reversine withdrawal, both myotube diameter and FI increased over time and were fully restored to the level of young myoblasts after 12 days (Figure 7a–c; myotube diameter, 4 days: 61 ± 21 μm, 8 days: 88 ± 32 μm, 12 days: 97 ± 30 μm; FI, 4 days: 40 ± 3%, 8 days: 53 ± 5%, 12 days: 60 ± 7%, *n* = 3, *p* < 0.001 as compared to SM).

We also investigated the effect of reversine on the dynamics of gene expression of key myogenic differentiation genes, including *MyoG, Mef2a*, and *Mef2c*, which are critical for myoblast fusion during skeletal muscle development (Bryantsev et al., 2012; Ganassi et al., 2018). To this end, myoblasts were coaxed to differentiate when they reached confluence (Day 0), and gene expression was measured over time using qRT‐PCR. For reversine‐treated cells, differentiation started 12 days after reversine withdrawal (Day 0). Our results showed that the expression of *MyoG, Mef2a*, and *Mef2c* was significantly increased in young cells from Day 1 of differentiation. Interestingly, in reversine‐treated cells, the expression of all three genes was delayed by 2 days but increased sharply thereafter, reaching similar (*MyoG, Mef2a*) or even higher (*Mef2c*) levels that those of young cells. Although *MyoG* and *Mef2a* remained at low levels in senescent myoblasts throughout the differentiation, *Mef2c* increased after two days but even in senescent myoblasts, it reached significantly higher levels in reversine‐treated cells (Figure 7d–f). These results suggested that reversine can improve myogenic differentiation capacity through increasing level of early differentiation markers, which are identified as essential regulators of skeletal muscle‐specific transcription.

### 2.8 Reversine improves heterochromatin modification and cellular metabolism of extreme DNA‐damage‐induced senescence

**FIGURE 8:**
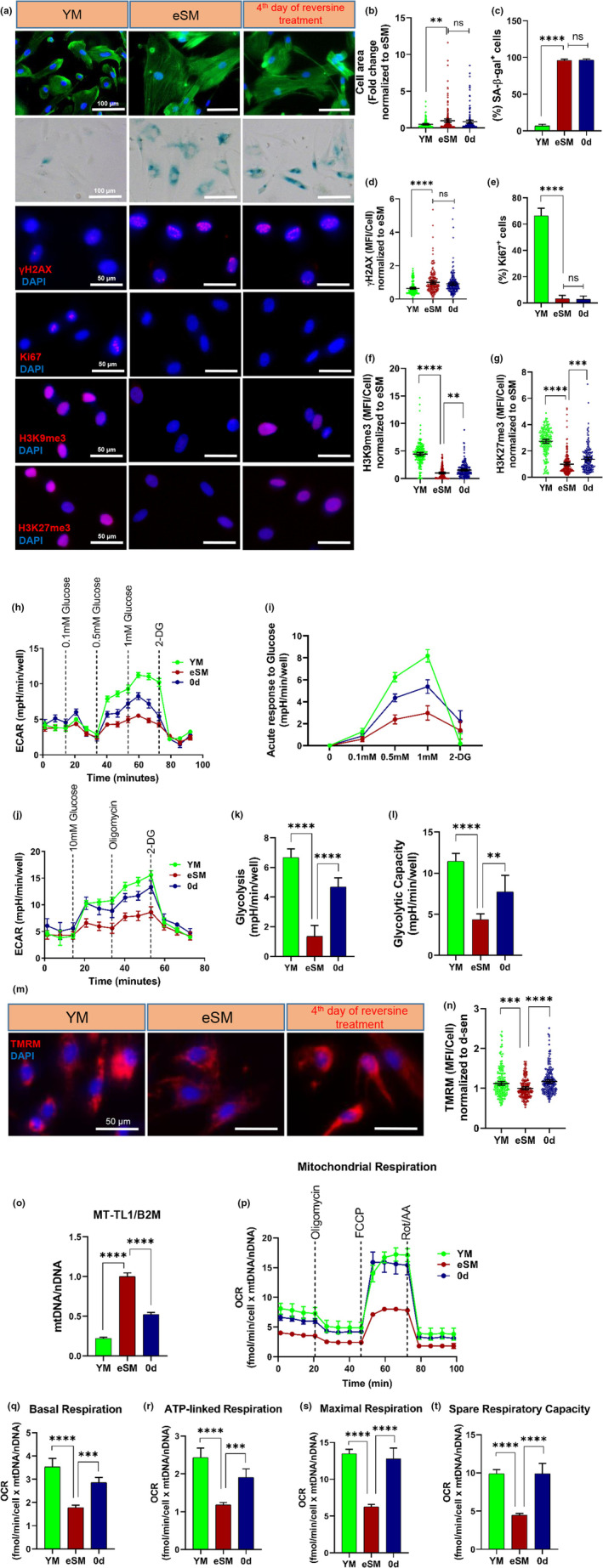
Reversine improves heterochromatin modification and cellular metabolism of DNA‐damage‐induced senescent myoblasts. (a) Immunostaining for F‐actin, SA‐β‐Gal, Ki67, γH2AX, H3K9me3, and H3K27me3 in young, etoposide‐treated myoblast (eSM), and eSM treated with reversine (scale bar = 100 μm for F‐actin, SA‐β‐Gal staining; scale bar = 50 μm for γH2AX, Ki67, H3K9me3 and H3K27me3, *n* = 150 cells) (b–g) Quantification of F‐actin, SA‐β‐Gal, γH2AX, Ki67, H3K9me3 and H3K27me3 positive myoblasts. (h) Measurements of extracellular acidification rate with different dosages of glucose. (i) Acute response indicating glucose sensitivity. (j–l) Measurements of extracellular acidification rate and calculation of glycolysis and glycolytic capacity from ECAR. (m and n) Representative images and quantification of tetramethylrhodamine methyl ester (TMRM) (scale bar = 50 μm, *n* = 150 cells). (o) Quantitative real‐time PCR quantification of mtDNA relative to nDNA (mtDNA/nDNA) as measured by primers specific to mitochondrial gene MT‐TL1 and nuclear gene Beta‐2‐Microglobulin (B2M). (p) Measurements of oxygen consumption rate (OCR) using Seahorse extracellular flux analyzer. (q–t) Calculations of the basal, maximal, reserved and ATP‐linked respiration rates. Data in OCR and ECAR plot is presented as mean ± SEM. Data in bar graphs are presented as mean ± SD and data in dot plots are presented as mean ± 95% confidence interval. * denotes *p* < 0.05, ** denotes *p* < 0.01, *** denotes *p* < 0.001, **** denotes *p* < 0.0001 and ns, not significant.

In addition to replicative senescence, we also induced senescence by acute and severe DNA damage using the chemical etoposide (Bang et al., 2019; Tamamori‐Adachi et al., 2018; Teng et al., 2021). In order to reach close to 100% senescent cells, young human myoblasts were treated with 50 μM etoposide for 24 h, followed by 48 h in fresh culture medium. This treatment resulted in almost 100% SA‐β‐gal+ cells (Figure 8a,c). In this acute model of senescence, treatment with reversine for 4 days had no effect on cell size, % SA‐β‐gal+ or γH2AX+ cells (Figure 8a–d) cells or proliferation (Figure 8a,e; Figure S6d). Nevertheless, it did increase the expression of heterochromatin marks H3K9me3 and H3K27me3, as well as glycolysis, mitochondrial function, and oxidative phosphorylation (Figure 8a,f–t). These results suggest that reversine could enhance heterochromatin modification and improve cellular metabolism of terminally senescent cells, experiencing severe DNA damage.

## 3 DISCUSSION

Recent work from our laboratory showed that the expression of a single pluripotency factor, NANOG, ameliorated many hallmarks associated with age‐related deterioration of myoblasts, including autophagy, energy homeostasis, genomic stability, nuclear integrity, and mitochondrial function (Shahini et al., 2021). NANOG expression also increased the number of muscle stem cells in a mouse model with no signs of tumorigenesis, demonstrating the feasibility of reversing cellular senescence in vitro and in vivo (Shahini et al., 2021). In this study, we report that the small molecule, reversine, can reverse several of the hallmarks of cellular senescence in human skeletal muscle cells. While several studies have shown that reversine increased cell plasticity and induced dedifferentiation to progenitor‐like state (Anastasia et al., 2006; Chen et al., 2004, 2007; Li et al., 2016), the effect of reversine on cellular senescence has not been reported yet. As previous work showed that cellular senescence in myoblasts does not depend on donor age but is strongly dependent on time in culture (Alsharidah et al., 2013; Decary et al., 1997; Rajabian et al., 2020), we used cells that were cultured for >30 population doublings as a model of cellular senescence. These cells exhibited all well‐known senescence hallmarks, including reduced proliferation, enlarged size and flattened morphology, DNA damage, mitochondrial dysfunction, impaired autophagy, and insulin resistance.

Treatment with reversine affected various cellular functions in a temporal sequence. For example, reversine quickly restored tri‐methylation of histone 3 at Lys9 and Lys27 (H3K9me3 and H3K27me3), which play crucial role in maintaining nuclear architecture and have been shown to decrease in senescent cells (Dillinger et al., 2017; Ito et al., 2018; Ocampo et al., 2016). Also, reversine decreased DNA damage, a hallmark of cellular senescence (López‐Otín et al., 2013), after only 4 days of treatment. On the contrary, SA‐β‐Gal expression, cell size and shape were restored 8–12 days after reversine treatment, indicating major reorganization of the actin cytoskeleton in agreement with previous work (Anastasia et al., 2006; Guo et al., 2021; Li et al., 2016). In agreement with previous reports where reversine was shown to inhibit cell proliferation (Huang et al., 2016; Park et al., 2019; Xia et al., 2021), cells did not proliferate during reversine treatment, likely due to inhibition of Aurora B kinase, but they started to proliferate after reversine was removed. Indeed, groups of small cells became apparent by Day 8 after reversine removal, and their number kept on increasing for up to 40 days. These results showed that restoring DNA damage and the state of heterochromatin preceded restoration of proliferation, SA‐β‐Gal expression, and cell size, prompting us to probe whether cellular functions such as metabolism or autophagy might be mediating the effects of reversine on revamping the function of senescent myoblasts.

In line with the existing literature (Baraibar et al., 2016; Pääsuke et al., 2016; Ravera et al., 2019; Refaie et al., 2006), senescent myoblasts showed reduced glycolysis and insulin resistance, stemming from impaired Akt2 signaling. Interestingly, reversine treatment for 4 days restored Akt2 signaling, glucose uptake, and insulin sensitivity. It also increased phosphorylation of AMPKα, a well‐known sensor of cellular energy that maintains cellular homeostasis through autophagy and modulates glycolysis and insulin sensitivity in the muscle (Herzig & Shaw, 2018). In addition to glucose metabolism, AMPK signaling is known to activate autophagy and improve mitochondrial health (Jeon, 2016). In agreement, we found that reversine induced autophagy in senescent cells as evidenced by increased LC3‐II and autophagy flux. Interestingly, reversine increased autophagy but it also decreased Akt phosphorylation in thyroid cancer cells (Lu et al., 2012). Inhibition of AMPK diminished the effect of reversine on mitochondrial function and oxidative phosphorylation, as well as glycolysis and glucose uptake, suggesting that autophagy activation might be necessary for improving mitochondrial health and restoring glycolysis and oxidative phosphorylation.

In addition, increased levels of Parkin and PINK1 suggested that reversine also enhanced mitophagy that was impaired in senescent cells. Indeed, reversine decreased mitochondrial content and restored mitochondrial membrane potential. It also improved the connectivity between glycolysis and oxidative phosphorylation, increasing the overall metabolic activity. Ultimately, the activation of autophagy and mitophagy in senescent cells might be necessary for the decreased cell size that was observed several days after treatment.

Our results showed that reversine could restore heterochromatin modifications, glycolysis, oxidative phosphorylation, and mitochondrial function with only a short 4‐day treatment, but the effects on proliferation were seen only several days after removal of reversine. This result suggested that the population of senescent cells might be heterogenous, with only a subset of cells being able to proliferate in response to reversine. To address this question, we employed etoposide to induce senescence by acute and extreme DNA damage, yielding 100% of SA‐β‐gal+ cells. Surprisingly, even under these conditions, reversine could restore the expression of heterochromatin marks H3K9me3 and H3K27me3, as well as glycolysis, mitochondrial function, and oxidative phosphorylation but did not restore cell proliferation. This may be the result of extreme DNA damage making it impossible for cells to re‐enter the cell cycle. It may also suggest that reversine could restore proliferation only in cells that were not terminally senescent. It would be very interesting to discover markers that distinguish between various stages of cellular senescence to identify the stage where proliferation can be restored by reversine.

Glycolysis, mitochondrial function, and the flow from glycolysis to the TCA cycle mediated by PDH play a crucial role in the myogenic differentiation capacity of myoblast cells (Hori et al., 2019; Tixier et al., 2013; Wagatsuma & Sakuma, 2013). Our results showed that senescent myoblasts lost their ability to form myotubes, but reversine treatment restored the myotube formation capacity. We demonstrated that reversine increased the level of early differentiation genes, such as *MyoG* and *MEF2*, which are essential regulators of skeletal muscle‐specific transcription (Bryantsev et al., 2012; Ganassi et al., 2018). As reversine treatment restored glycolysis, insulin sensitivity, mitochondrial membrane potential, the connectivity between glycolysis and TCA cycle, and expression of early myogenic differentiation markers, reversine‐treated cells restored myotube formation capacity several days after treatment.

Based on the temporal sequence that cellular functions were affected by reversine, it appears that reversine may first activate autophagy and mitophagy, leading to restored metabolic functions such as glycolysis and mitochondrial respiration within 4 days of treatment. These changes enhance the overall cellular bioenergetics, setting the stage for further changes, including actin reorganization, reduction in cell size, increased proliferation and restoration of myofiber formation capacity, ultimately restoring the function of senescent muscle cells. Cellular reprogramming using the four Yamanaka factors (OSKM) has also been employed as a strategy for cellular rejuvenation in vitro and in vivo (Takahashi et al., 2007). Although a lot can be learned about the biology of aging using this approach, translating OSKM reprogramming into therapy remains a major challenge (de Magalhães & Ocampo, 2022), mostly due the high likelihood of teratoma formation, even when used intermittently (Abad et al., 2013). Alternatively, senolytics have been shown to selectively eliminate senescent cells restoring the function of aging tissues (Kirkland & Tchkonia, 2020; Zhu et al., 2015), but they do so in a cell and tissue‐specific manner (Niedernhofer & Robbins, 2018), suggesting that although some drug combinations may enhance the function of some tissues, they may impart unwanted side effects on others. Here, we show that treating senescent myoblasts with a single small molecule, reversine, for 4 days ameliorated many aspects of cellular senescence, restoring metabolism, and myofiber formation capacity by activating autophagy. Since reversine is already being investigated as an anticancer drug (Lu et al., 2012; Park et al., 2019; Piccoli et al., 2019), our results suggest that it may have the potential for clinical translation to enhance the function of aging tissues and improve health span.

## 4 MATERIALS AND METHODS

### 4.1 Cell culture

Human myoblasts were purchased from Cook MyoSite (Pittsburgh, PA). Cells were isolated from quadriceps muscle of three donors (18‐year‐old male, 25‐year‐old female, and 75‐year‐old female). The cells were cultured on Matrigel (0.1 mg/ml; CORNING, Corning, NY)‐coated T175 flasks and expanded in skeletal muscle cell growth medium (GM) as described previously (Rajabian et al., 2020). The cells cultured for <5 passages (<7 population doubling, termed young myoblast or YM) and p > 10 passages (> 20 population doubling, termed senescent myoblast or SM). The cells were cultured in a humidified incubator at 37°C and 10% CO_2_, and the medium was replenished every other day. Cells were passaged every 4–5 days before they reached 80% confluence.

The human myoblasts were cultured for more than 10 passages (equivalent to >50 days of culture) to show well‐known hallmarks of cellular senescence as described previously (Rajabian et al., 2020; Shahini et al., 2021). These senescent cells were treated with reversine (Sigma‐Aldrich Chemical Company) at final concentration of 5 μM in GM. As reversine is dissolved in dimethylsulfoxide (DMSO), the control cells were cultured in the same volume of DMSO without reversine. To block AMPK activity, Compound C (CC; Cayman Chemical, Ann Arbor, MI, catalog no. 11967) was added to the GM for at least 18 h prior to experiments. To differentiate myoblasts into multinucleated myotubes, myoblasts were seeded on Matrigel (0.1 mg/ml)‐coated dishes and allowed to reach >90% confluence in GM. Then, the cells were switched to differentiation medium composed of high‐glucose DMEM supplemented with insulin (10 μg/ml), epidermal growth factor (10 ng/ml), BSA (500 μg/ml), and gentamicin (50 μg/ml) for a period of 7 days. For reversine‐treated cells, reversine was removed for 0, 4, 8, and 12 days prior to differentiation.

### 4.2 Etoposide treatment to induce cellular senescence

Young human myoblasts were treated with 50 μM etoposide (E1383, Sigma‐Aldrich, St. Louis, MO) for 24 h, followed by 48 h in fresh culture medium. This concentration of etoposide induced almost 100% positive for SA‐β‐Gal.

### 4.3 Measurement of cell radius

To measure the cellular radius, the cells were detached from the surface using 0.25% Trypsin–EDTA and centrifuged at 300*g* for 5 min. The cell pellet was resuspended in PBS, and 10 μl was inserted in hemocytometer. Phase images were acquired using EVOS FL inverted digital microscope (ThermoFisher Scientific), and the cellular area was measured using the ImageJ software. Cell radius was calculated using the following equation: area = π × *R* ^2^ (Morgan et al., 2016).

### 4.4 Senescence‐associated‐β‐galactosidase

The SA‐β‐Gal activity was detected using the Senescence Detection Kit (ab65351, Abcam) according to the manufacturer's instructions. Cells were photographed using the Zeiss Axio Observer Z1a microscope and a 10 × objective (Plan‐APOCHROMAT). The number of SA‐β‐Gal‐positive and total cells were counted in five randomly selected fields of view (total of >250 cells were counted per sample).

### 4.5 mtDNA content quantification

DNA was isolated using the QIAmp DNA Mini Kit (QIAGEN, Germantown, MD catalog no. 51304) according to the manufacturer's instructions. Quantitative real‐time PCR was performed using the SYBR Green Kit (Bio‐Rad, Hercules, CA, catalog no. 172–5120) with 25 ng of DNA used per reaction. mtDNA was quantified using the following human primers for mitochondrially encoded tRNALeu (UUR) gene (MT‐TL1, forward primer: 5′‐CACCCAAGAACAGGGTTTGT‐3′ and reverse primer: 5′‐TGGCCATGGGTATGTTGTTA‐3′), and nDNA was quantified using the following human primers for Beta‐2‐Microglobulin (B2M) gene [forward primer: 5′‐GAGGCTATCCAGCGTACTCCA‐3′ and reverse primer: 5′‐CGGCAGGCATACTCATCTTTT‐3′], respectively. Both mtDNA and nDNA threshold cycle average values were obtained, and the mtDNA content was calculated relative to nDNA, mtDNA/nDNA = 2^(CTnDNA−CTmtDNA)^.

### 4.6 Seahorse assay

We used Seahorse extracellular flux (XFe96) analyzer (Agilent technologies, Santa Clara, CA) to measure the extracellular acidification rate, which is a measure of glycolysis. The myoblast cells were seeded at a density of 3000 cell/cm^2^ on matrigel‐coated XFe96 seahorse culture plates for 12 h. At the time of the assay, the cells were washed and culture medium was changed to seahorse assay medium for 45 min (XF DMEM medium, Cat No. 103575‐100, Agilent technologies, Santa Clara, CA), and glycolysis and glycolytic capacity were measured after sequential injection of 10 mM glucose, 1 μM oligomycin, and 10 mM 2‐DG. All calculations were based on Agilent Seahorse XF Technology white paper document or manufacturer protocol. Acute response (glucose sensitivity) was measured after different dosage of glucose (0.1, 0.5 and 1 mM) and is equal to [Max ECAR after glucose injection‐ Min ECAR before injection].

We also measured the oxygen consumption rate (OCR) and performed mito stress test. The cells were seeded at a density of 10,000 cell/cm^2^ on matrigel‐coated XFe96 seahorse culture plates for 12 h. At 1.5 h prior to the assay, the cells were incubated in XF base medium (Agilent) supplemented with 10 mM glucose, 1 mM pyruvate, and 2 mM glutamine. Subsequently, OCR was measured after the addition of 1 μM oligomycin, 1.5 μM FCCP, and a mixture containing 0.5 μM each of antimycin A and rotenone. After the seahorse measurements were completed, total cellular content was measured using a CyQUANT Cell Proliferation Assay kit (ThermoFisher Scientific, catalog no. C7026), and the OCR values were normalized to (number of cells × mtDNA/nDNA).

### 4.7 Adenosine triphosphate measurement assay

Myoblasts were seeded on Matrigel (0.1 mg/ml)‐coated 48‐well plates (5 × 10^3^ cell/cm^2^). ATP concentration was measured per manufacturer's instructions. In brief, 50 μl of detergent was added to each sample for 5 min to lyse the cells under continuous mixing in an orbital shaker at 600–700 rpm. Then, 50 μl of substrate solution was added to cell lysate and incubated for 5 min in an orbital shaker at 600–700 rpm. Hundred microliters of each lysate was transferred to a well in a 96‐well plate, and luminescence was recorded using a Biotek Synergy 4 plate reader as directed by the manufacturer (background luminescence of empty wells was subtracted from each value).

### 4.8 Western blots

Myoblast cells were lysed in buffer containing 62.5 mM Tris–HCl (pH 6.8 at 25 °C), 2% (w/v) SDS, 10% (v/v) glycerol, 0.1% (w/v) bromophenol blue, 41.67 mM dithiothreitol (DTT) (Cell Signaling, Danver, MA), and protease inhibitor cocktail (Sigma‐Aldrich, St. Louis, MO). The protein concentration was determined using the Bradford assay (Bradford, 1976). Lysates were denatured by incubation at 95°C for 5 min, and proteins were loaded at 45 μg per lane and were separated in 12% acrylamide gels (Waltham, MA) by SDS‐polyacrylamide gel electrophoresis based on their molecular weight. After transferring proteins to nitrocellulose membranes (Bio‐Rad) using the Trans‐Blot Turbo Transfer System (Bio‐Rad, Hercules, California), the membranes were blocked 5% (w/v) nonfat dry milk in Tris‐buffered saline with 0.1% Tween® 20 detergent (TBST) buffer (20 mM tris, 150 mM NaCl, and 0.1% Tween 20) for 1 h at room temperature. Subsequently, membranes were incubated overnight at 4°C with antibodies including, Akt1 (Clone No. 9Q7, 1:1000 dilution in blocking buffer, Invitrogen), pAkt1‐ Ser473 (Clone No. 14–6, 1:1000 dilution in blocking buffer, Invitrogen), Akt2 (Clone No. 4H7, 1:1000 dilution in blocking buffer, Invitrogen), pAkt2‐Ser474 (Cat No. PA5‐104870, 1:1000 dilution in blocking buffer, Invitrogen), LC3A/B (Clone No. D3U4C, Cat No. 12741, Cell Signaling Technology), AMPKα (Cat No. 2532, 1:1000 dilution in blocking buffer, Cell Signaling Technology), pAMPKα (Thr172, Clone No. 40H9, Cat No. 2535, 1:1000 dilution in blocking buffer, Cell Signaling Technology), Parkin (Cat No. 2132, 1:1000 dilution in blocking buffer, Cell Signaling Technology), PINK1 (Clone No. D8G3, Cat No. 6946, 1:1000 dilution in blocking buffer, Cell Signaling Technology), PDH (Cat No. ab126203, 1:1000 dilution in blocking buffer; Abcam, Cambridge, MA), pPDH‐E1α (pSer^232^) (Cat No. AP1063, 1:1000 dilution in blocking buffer; Millipore, Billerica, MA), and glyceraldehyde 3‐phosphate dehydrogenase (GAPDH, Clone No. 14C10, Cat No. 2118, 1:10,000 dilution in blocking buffer; Cell Signaling). Finally, the protein bands were visualized using horseradish peroxidase conjugated secondary antibodies and a chemiluminescence kit (Cell Signalling, Danvers, MA) according to the manufacturer's instructions. Luminescent blots were imaged using ChemiDoc™ Touch Imaging System (Bio‐Rad, Hercules, CA).

### 4.9 Immunocytochemistry

Myoblasts were fixed in 10% formalin for 10 min at RT. The fixed cells were permeabilized with 0.1% (v/v) Triton X‐100/PBS for 10 min at RT and blocked with blocking buffer [5% (v/v) goat serum in 0.01% (w/v) Triton X‐100/PBS] at RT for 1 h. Next, samples were immunostained with antibodies against myosin heavy chain (MYHC; Clone No. A4.1025, 1:500 dilution in blocking buffer; Millipore, Billerica, MA), sarcomeric alpha actinin (SAA; Clone No. EP2529Y, 1:200 dilution in blocking buffer; Abcam), γH2AX (Clone No. S139, 1:200 in blocking buffer; Cell Signaling Technology), H3K9me3 (Cat No. ab8898, 1:500 in blocking buffer; Abcam), H3K27me3 (Clone No. C36B11, 1:200 in blocking buffer; Cell Signaling Technology), desmin (Clone No. D93F5, 1:100 in blocking buffer; Cell Signaling Technology), Ki67 (Cat No. ab15580, 1:200 in blocking buffer; Abcam), F‐Actin (Alexa Fluor 488 Phalloidin, Thermo Fisher Scientific; 1:100 dilution in PBS with 1% (w/v) Bovine Serum Albumin for 2 h). Subsequently, the cells were stained with Alexa Fluor 568 or 488 conjugated goat antimouse or goat antirabbit antibodies (1:250 dilution in blocking buffer; ThermoFisher Scientific) and counterstained with Hoechst 33342 nuclear dye for 5 min (1:1000 dilution in PBS, Thermo Fisher Scientific).

TMRM (ThermoFisher Scientific) and MitoTracker Red CMXRos (ThermoFisher Scientific) were used to monitor the mitochondrial membrane potential. The cells were stained by the addition of these dyes to the culture medium at 100 nM for 30 min at 37°C. The cells were stained with the Hoechst 33342 nuclear dye for 5 min (1:1000 dilution in PBS; ThermoFisher Scientific). Cells were imaged using Zeiss Axio Observer Z1 (LSM 510; Zeiss, Oberkochen, Germany) equipped with digital camera (ORCA‐ER C4742‐80; Hamamatsu, Bridgewater, NJ).

### 4.10 Statistical analysis

Statistical analysis was performed using one‐way or two‐way analysis of variance (ANOVA) analysis followed by Tukey's multiple comparisons test using GraphPad Prism version 8 software. Each experiment was repeated three times with at least triplicate samples in each experiment. Data were reported as mean ± standard deviation (SD). Statistical significance was denoted as *p < 0.05, **p < 0.01, ***p < 0.001, ****p < 0.0001, and ns, not significant.

## AUTHOR CONTRIBUTIONS

Experiments were planned and designed by Nika Rajabian and Stelios T. Andreadis. Experimental data were generated and collected by Nika Rajabian, Izuagie Ikhapoh, Debanik Choudhury, Shilpashree Saha, Aishwarya Surendra Kalyankar, Aref Shahini, Kendall Breed, and Pihu Mehrotra. Data analysis and interpretation were performed by Nika Rajabian and Stelios T. Andreadis. Writing and critical revisions of the manuscript were performed by Nika Rajabian and Stelios T. Andreadis.

## CONFLICT OF INTEREST

The authors declare that they have no conflict of interest.

## Supporting information


Figures S1

FigureCaptions

## Data Availability

The data that support the findings of this study are openly available in Mendeley Data at https://data.mendeley.com/datasets/wbymrhr5g7/1, DOI:10.17632/wbymrhr5g7.1.
