# Supplementary material for: Reversine ameliorates hallmarks of cellular senescence in human skeletal myoblasts via reactivation of autophagy
Source: Aging Cell. 2023 Jan 10;22(3):e13764. doi: 10.1111/acel.13764 (PMC10014065; doi:10.1111/acel.13764)
Supplement: Supplementary file 1 — Figures S1 [file ACEL-22-e13764-s001.pptx]

## Slide 1
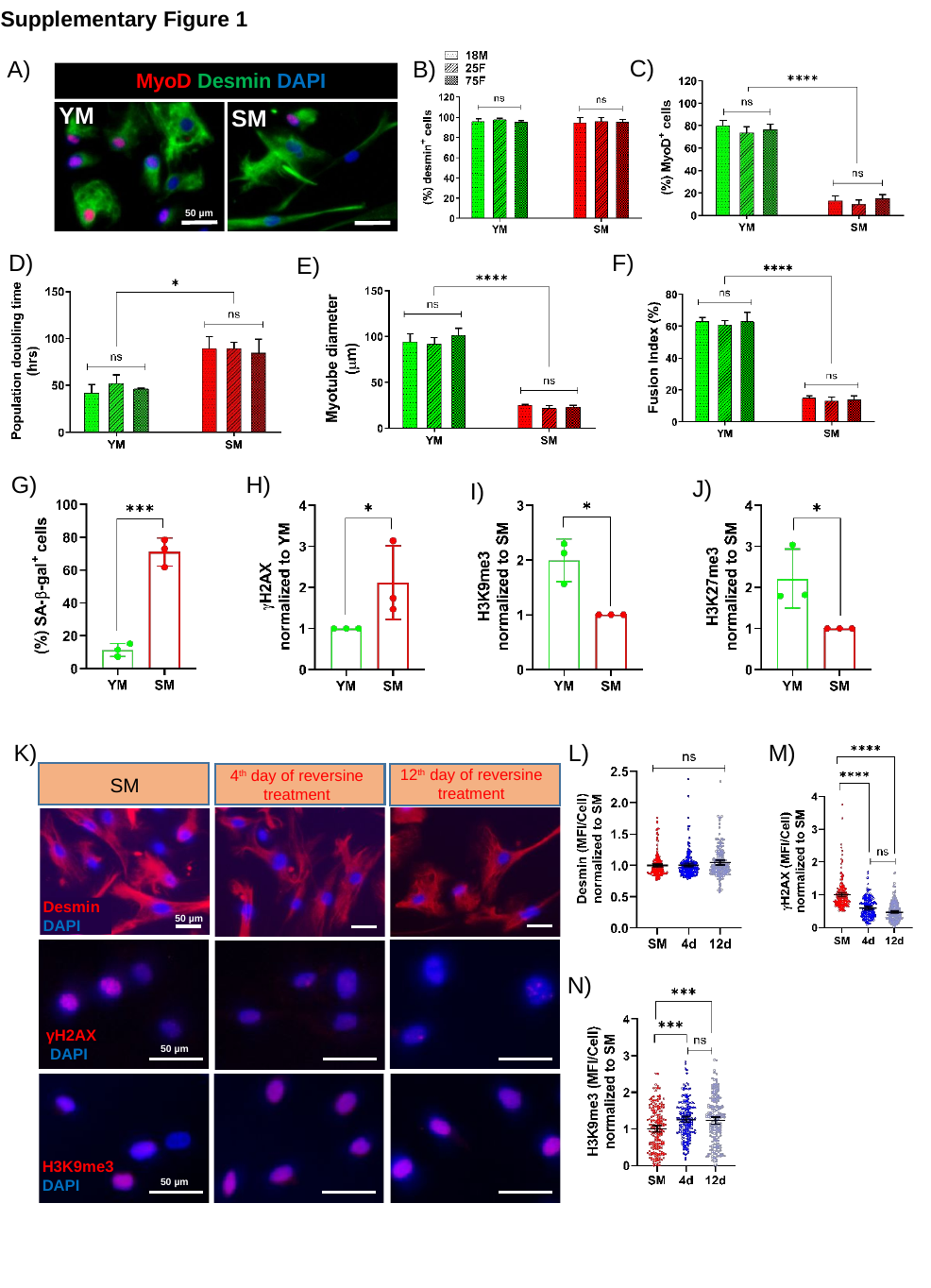

Supplementary Figure 1
C)
B)
A)
MyoD Desmin DAPI
YM
SM
50 µm
F)
D)
E)
H)
G)
J)
I)
K)
L)
M)
12th day of reversine treatment
4th day of reversine treatment
SM
Desmin
DAPI
50 µm
N)
γH2AX
 DAPI
50 µm
H3K9me3
DAPI
50 µm

## Slide 2
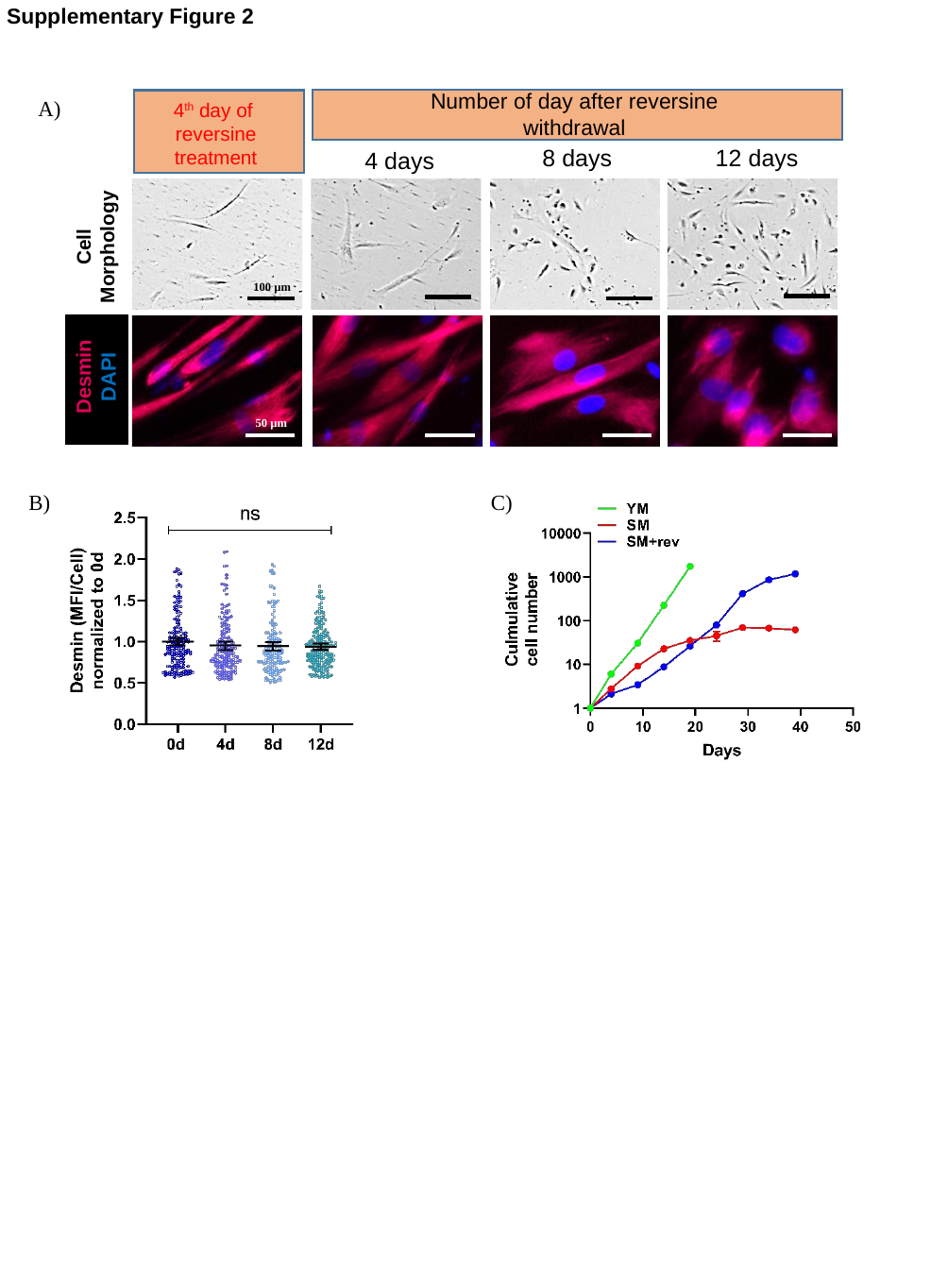

Supplementary Figure 2
Number of day after reversine withdrawal
A)
4th day of
reversine treatment
8 days
12 days
4 days
Cell Morphology
100 µm
Desmin
DAPI
50 µm
B)
C)

## Slide 3
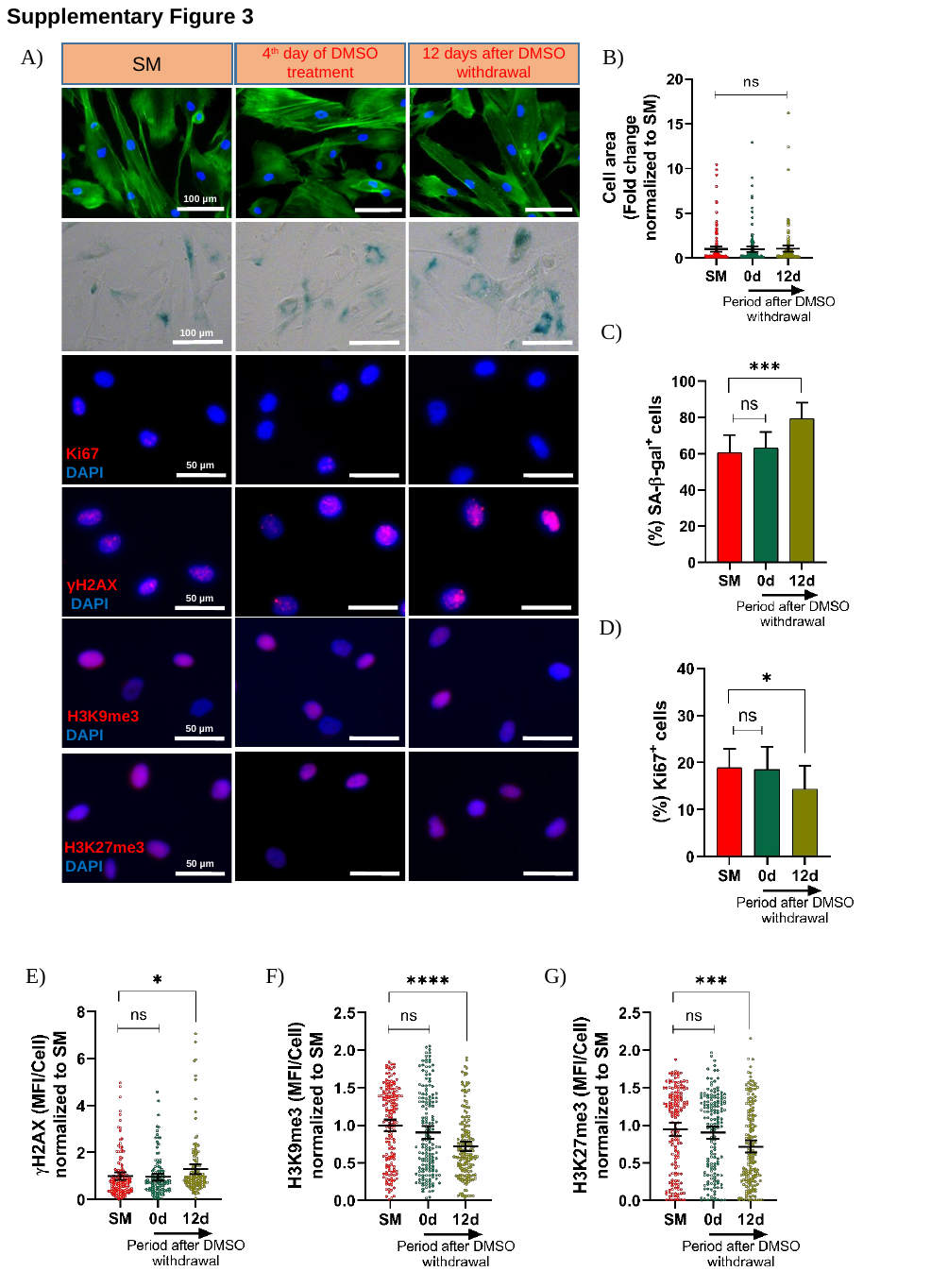

Supplementary Figure 3
A)
4th day of DMSO treatment
12 days after DMSO withdrawal
B)
SM
100 µm
C)
100 µm
Ki67
DAPI
50 µm
γH2AX
 DAPI
50 µm
D)
H3K9me3
DAPI
50 µm
H3K27me3
 DAPI
50 µm
E)
F)
G)

## Slide 4
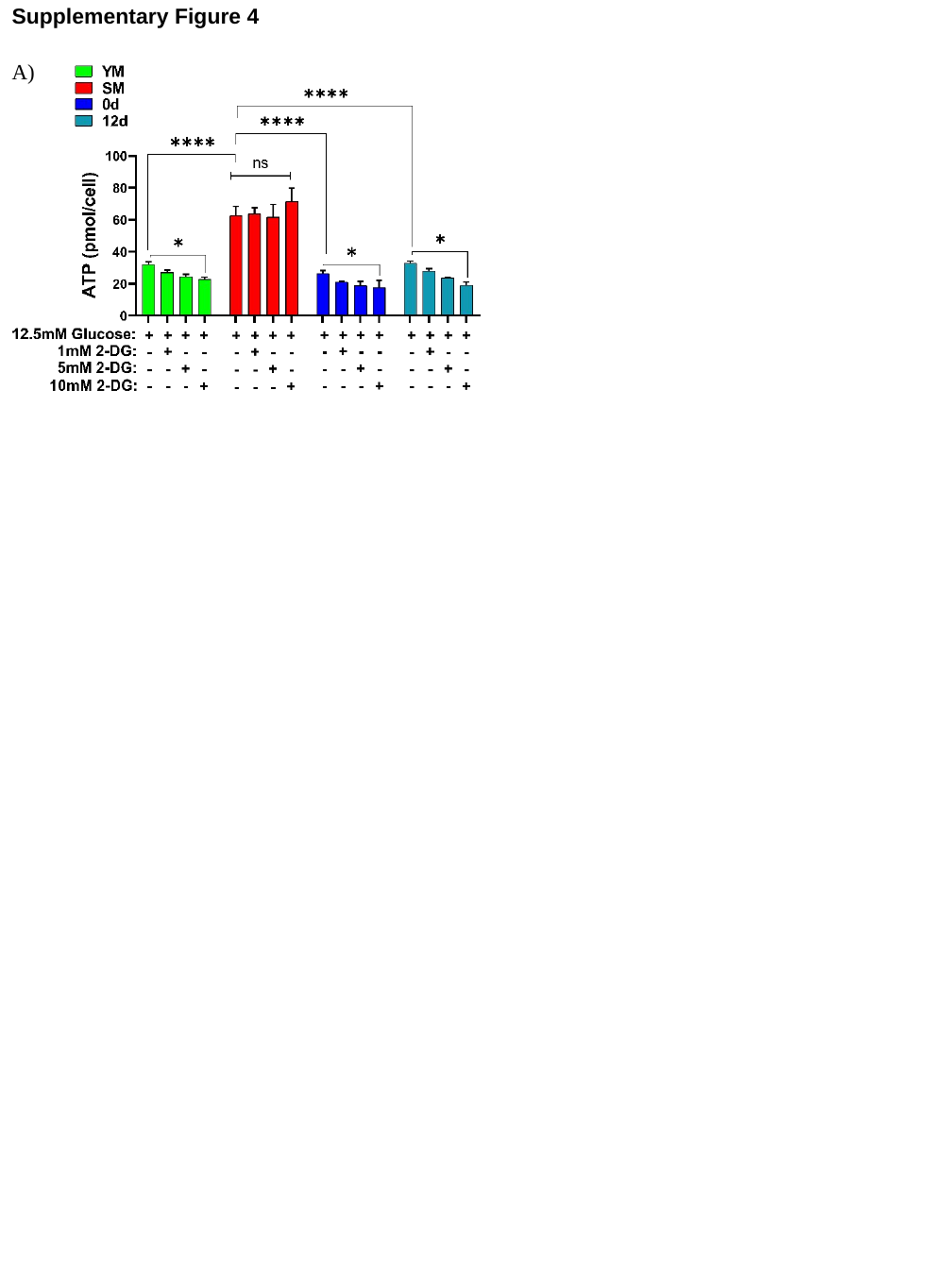

Supplementary Figure 4
A)

## Slide 5
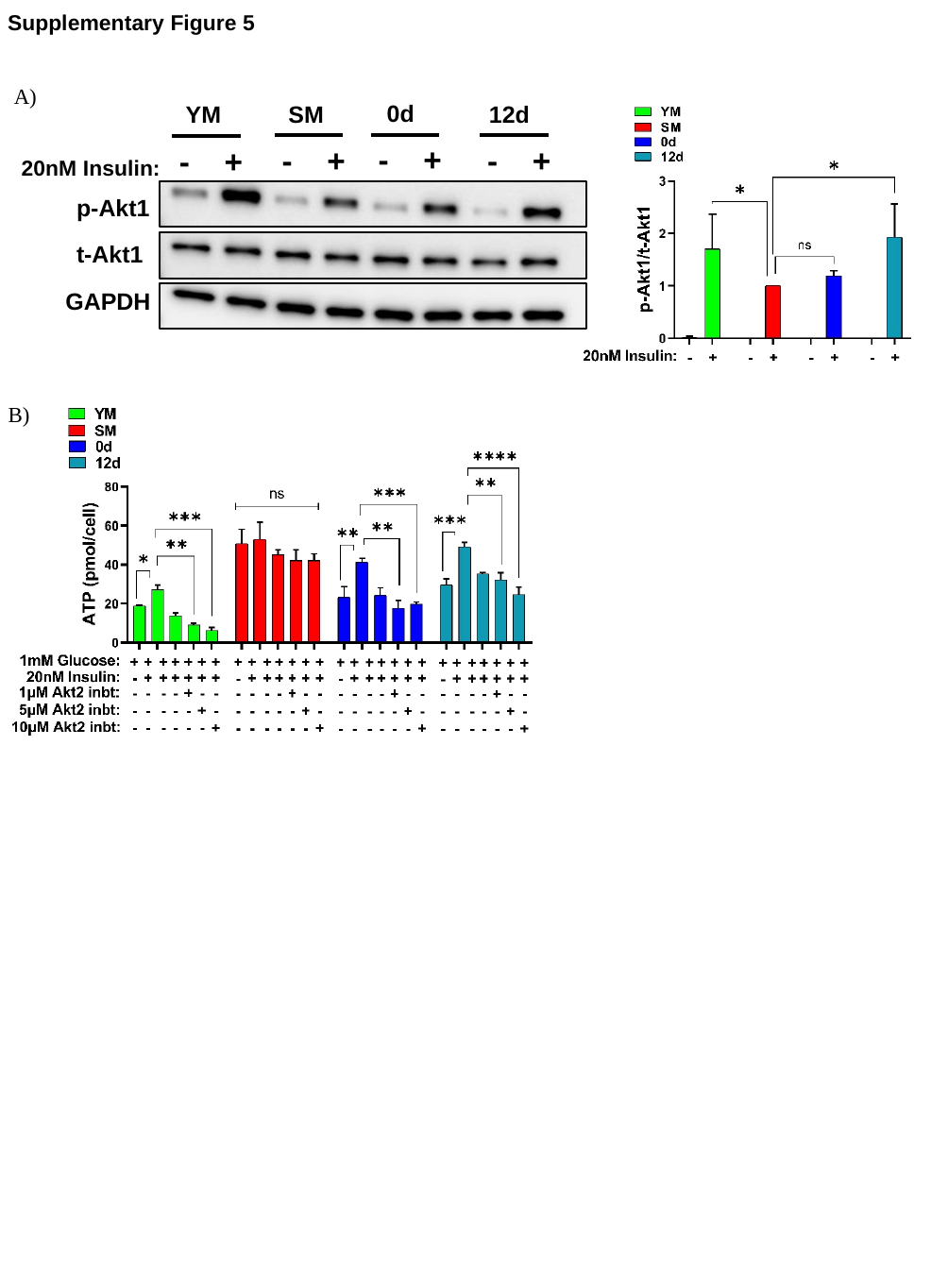

Supplementary Figure 5
A)
0d
SM
12d
YM
- +
- +
- +
- +
20nM Insulin:
p-Akt1
t-Akt1
GAPDH
B)

## Slide 6
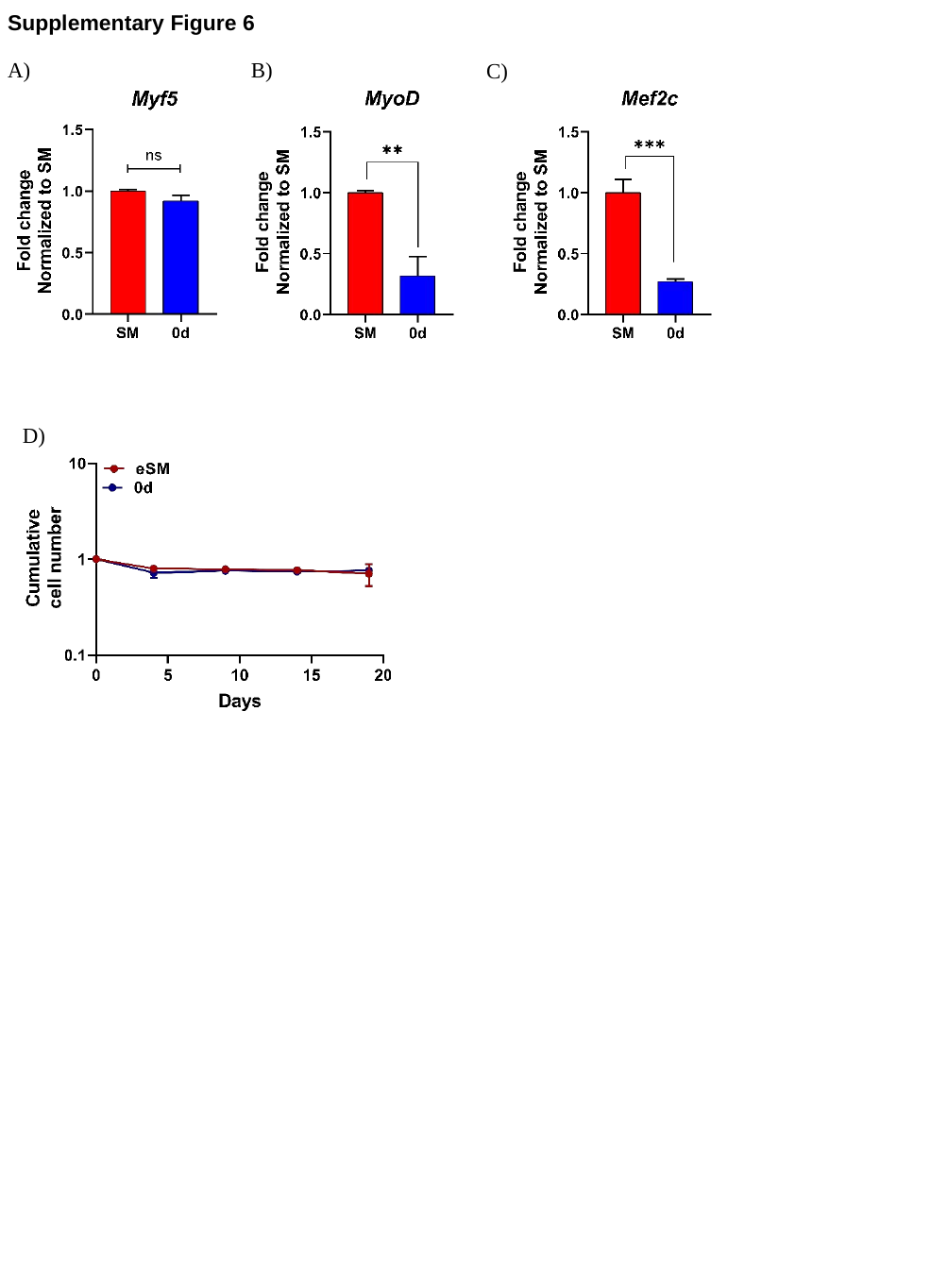

Supplementary Figure 6
A)
B)
C)
D)
