# Supplementary material for: Reversine ameliorates hallmarks of cellular senescence in human skeletal myoblasts via reactivation of autophagy
Source: Aging Cell. 2023 Jan 10;22(3):e13764. doi: 10.1111/acel.13764 (PMC10014065; doi:10.1111/acel.13764)
Supplement: Supplementary file 2 — FigureCaptions [file ACEL-22-e13764-s002.docx]

**Figure S1. (A-C)** Immunostaining and quantification of Desmin and MyoD in young and senescent myoblasts (donors: 18M, 25F and 75F) (scale bar= 50µm, n=150 cells). **(D)** Population doubling times were calculated for young and senescent human myoblasts. **(E, F)** The average diameter of myotubes and fusion index (number of myonuclei/total number of nuclei × 100%). **(G-J)** Quantification of SA-β-Gal, γH2AX, H3K9me3 and H3K27me3 positive cells in young and senescent myoblasts from 3 different donors. Each dot in the bar graph indicates one donor. (K-N) Immunostaining and quantification of Desmin, γH2AX, and H3K9me3 in senescent myoblasts, control or treated with reversine for 4 days or 12 days (scale bar=50µm, n=150 cells). Data in bar graphs are presented as mean ± std and data in dot plots are presented as mean ± 95% CI. * denotes *p* < 0.05, *** denotes p < 0.001 and **** denotes p < 0.0001, and ns, not significant.

**Figure S2.** (**A, B**) Phase images immunostaining and quantification for desmin in senescent cells at 0, 4, 8, 12 days post reversine treatment. Cells were treated for reversine for 4 days. (**C**) Cumulative cell number in young and senescent control of reversine treated myoblasts at the indicated times; data shown as means ± SD.

**Figure S3. (A)** Immunostaining for F-actin, SA-β-Gal, Ki67, γH2AX, H3K9me3 and H3K27me3 in senescent myoblasts immediately (d0) or 12 days (d12) after DMSO treatment. (scale bar=100µm for F-actin, SA-β-Gal staining; scale bar=50µm for Ki67, γH2AX, H3K9me3 and H3K27me3 staining, n=150 cells) **(B-G)** Quantification of F-actin, SA-β-Gal, Ki67, γH2AX, H3K9me3 and H3K27me3 in senescent myoblast on d0 and d12 post DMSO treatment.

**Figure S4. (A)** The levels of ATP level measured in the presence of the indicated concentrations of 2-DG (hexokinase inhibitor, IC50=5mM) in 12.5mM glucose containing medium. Data in ECAR plot is presented as mean ± SEM and data in bar graphs are presented as mean ± std. * denotes *p* < 0.05, **** denotes *p* < 0.0001 and ns, not significant.

**Figure S5. (A**) Western blots and quantification of total (t) and phosphorylated (p)Akt2 in young and senescent control or reversine treated myoblasts on 0d or 12d; GAPDH served as a loading control. (**B**) The levels of intracellular ATP upon treatment with 12.5mM glucose, 20mM insulin and Akt2 inhibitor at the indicated concentrations. Data in bar graphs are presented as mean ± std. * denotes *p* < 0.05, ** denotes *p* < 0.01, *** denotes *p* < 0.001, **** denotes *p* < 0.0001 and ns, not significant.

**Figure S6. (A-C)** Quantitative real-time PCR quantification of gene expression of *Myf5, MyoD* and *Mef2c* in senescent control and reversine treated myoblasts. **(D)** Cumulative cell number of etoposide treated senescent myoblasts (eSM) and reversine treated eSM over time in culture; data shown as means ± SD. Data in bar graphs are presented as mean ± std. ** denotes *p* < 0.01, *** denotes *p* < 0.001, and ns, not significant.
